# The Landscape of Inappropriate Laboratory Testing: A 15-Year Meta-Analysis

**DOI:** 10.1371/journal.pone.0078962

**Published:** 2013-11-15

**Authors:** Ming Zhi, Eric L. Ding, Jesse Theisen-Toupal, Julia Whelan, Ramy Arnaout

**Affiliations:** 1 Harvard Medical School, Boston, Massachusetts, United States of America; 2 Channing Laboratory, Department of Medicine, Brigham and Women's Hospital, Boston, Massachusetts, United States of America; 3 Department of Nutrition, Harvard School of Public Health, Boston, Massachusetts, United States of America; 4 Division of General Medicine and Primary Care, Department of Medicine, Beth Israel Deaconess Medical Center, Boston, Massachusetts, United States of America; 5 Countway Library of Medicine, Harvard Medical School, Boston, Massachusetts, United States of America; 6 Department of Pathology, Beth Israel Deaconess Medical Center, Boston, Massachusetts, United States of America; 7 Division of Clinical Informatics, Department of Medicine, Beth Israel Deaconess Medical Center, Boston, Massachusetts, United States of America; Gentofte University Hospital, Denmark

## Abstract

**Background:**

Laboratory testing is the single highest-volume medical activity and drives clinical decision-making across medicine. However, the overall landscape of inappropriate testing, which is thought to be dominated by repeat testing, is unclear. Systematic differences in initial vs. repeat testing, measurement criteria, and other factors would suggest new priorities for improving laboratory testing.

**Methods:**

A multi-database systematic review was performed on published studies from 1997–2012 using strict inclusion and exclusion criteria. Over- vs. underutilization, initial vs. repeat testing, low- vs. high-volume testing, subjective vs. objective appropriateness criteria, and restrictive vs. permissive appropriateness criteria, among other factors, were assessed.

**Results:**

Overall mean rates of over- and underutilization were 20.6% (95% CI 16.2–24.9%) and 44.8% (95% CI 33.8–55.8%). Overutilization during initial testing (43.9%; 95% CI 35.4–52.5%) was six times higher than during repeat testing (7.4%; 95% CI 2.5–12.3%; *P* for stratum difference <0.001). Overutilization of low-volume tests (32.2%; 95% CI 25.0–39.4%) was three times that of high-volume tests (10.2%; 95% CI 2.6–17.7%; *P*<0.001). Overutilization measured according to restrictive criteria (44.2%; 95% CI 36.8–51.6%) was three times higher than for permissive criteria (12.0%; 95% CI 8.0–16.0%; *P*<0.001). Overutilization measured using subjective criteria (29.0%; 95% CI 21.9–36.1%) was nearly twice as high as for objective criteria (16.1%; 95% CI 11.0–21.2%; *P* = 0.004). Together, these factors explained over half (54%) of the overall variability in overutilization. There were no statistically significant differences between studies from the United States vs. elsewhere (*P* = 0.38) or among chemistry, hematology, microbiology, and molecular tests (*P* = 0.05–0.65) and no robust statistically significant trends over time.

**Conclusions:**

The landscape of overutilization varies systematically by clinical setting (initial vs. repeat), test volume, and measurement criteria. Underutilization is also widespread, but understudied. Expanding the current focus on reducing repeat testing to include ordering the right test during initial evaluation may lead to fewer errors and better care.

## Introduction

Laboratory testing is an integral part of modern medicine. Testing figures prominently across specialties and in multiple medical contexts, including outpatient screening (e.g. cholesterol for heart disease, hemoglobin A1c for diabetes mellitus), inpatient diagnosis and management, and disease monitoring (e.g. tumor markers for cancer). As a result, testing is the single highest-volume medical activity, with an estimated 4–5 billion tests performed in the United States each year [1]. Testing is often the principal basis for more costly downstream care. It also features prominently in pay-for-performance guidelines and compliance standards, making it a potential target for cost savings under global payment plans [2]–[5]. However, the prevalence of inappropriate testing is unknown.

Inappropriate testing takes several forms. *Overutilization* or over-ordering refers to tests that are ordered but not indicated, while *underutilization* refers to tests indicated but not ordered. There is inappropriate *initial* testing, for example, during the initial evaluation of a patient or in response to new signs or symptoms, and inappropriate *repeat* testing. There are also different kinds of inappropriateness criteria. *Objective* criteria are clearly defined and investigator-independent, while *subjective* criteria depend on expert review. *Restrictive* criteria require there to be a clear indication for ordering a test, while *permissive* criteria require only that there be no contraindication. Restrictive and permissive criteria – terms we coin – respectively represent “guilty-until-proven-innocent” and “innocent-until-proven-guilty” approaches to inappropriate testing.

Whatever the context or criteria, inappropriate testing can cause harm and lead to medical errors. Overutilization can result in unnecessary blood draws and other sample-collection procedures [6], [7]. It also increases the likelihood of false-positive results, which can lead to incorrect diagnoses, increased costs, and adverse outcomes due to unwarranted additional intervention [8], [9]. Underutilization can result in morbidity due to delayed or missed diagnoses and in downstream overutilization. Over- and underutilization can both lead to longer hospital stays and contribute to legal liability. A recent review of malpractice claims in an outpatient setting found failures to order or correctly interpret laboratory tests in one in every eight claims, often with multiple occurrences per claim [10]; a similar study from an emergency-room setting found a rate of one in seven [11].

Studies of specific tests and clinical scenarios suggest that inappropriate laboratory testing is a serious problem throughout medicine [12]–[20]. However, there are also studies that have found rates of inappropriate testing to be low [21]–[28]. Recent trends in medicine can be marshaled to support either view. On the one hand, evidence-based practice and clinical decision support (CDS) encourage appropriate testing. On the other, defensive medicine and panel-based ordering encourage overutilization [29]. The only previous systematic review, published 15 years ago, can likewise be interpreted variably: it found rates of inappropriate testing that ranged from 4% to 95% [27]. However, it covered only through September 1997 and thus preceded major recent developments in health-care quality [30]–[34]. To chart the landscape of inappropriate testing, we performed a systematic review of audits on the appropriateness of laboratory testing over the past 15 years in order to estimate the overall prevalence of inappropriate laboratory testing and compared over- vs. underutilization, inappropriate initial vs. repeat testing, and different types of criteria.

## Materials and Methods

### Methodology

We conducted our analysis according to MOOSE and PRISMA guidelines (see Checklist S1 and Checklist S2 in File S1) [35]. No prior protocol existed for our study. The context was global.

### Data Sources and Searches

We searched Medline for studies published between October 1997 and January 2012 by crossing relevant medical subject headings (MeSH terms) with subheadings and text words (e.g. “utilization,” “laboratory test (s)”). Only citations on human subjects were included. For completeness, we repeated the search without subheadings and combined the results of these two searches. We similarly searched the Embase (Elsevier), BIOSIS (Thomson Reuters), CINAHL (EBSCO), and Cochrane databases. See File S1 for details.

### Study Selection

**Figure 1:**
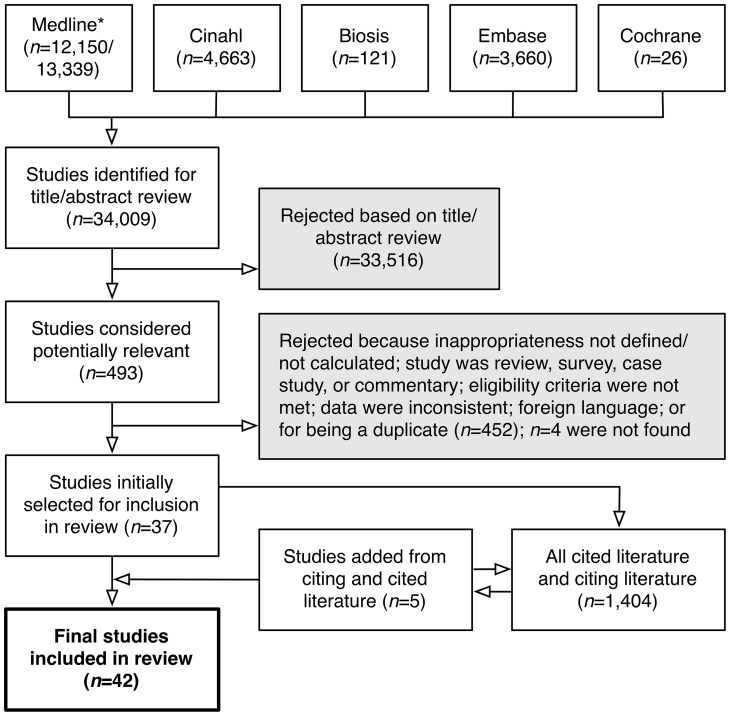
Literature Search Strategy and Results, 1997–2012. The indicated databases were searched as described in the main text and File*Results from searching with and without subheadings.

For each citation, two investigators [M.Z. and R.A.] independently screened the title and abstract for potential relevance. The results were combined. For citations considered potentially relevant, we evaluated the study in depth according to the following specific inclusion and exclusion criteria. Studies were included if (i) they specified valid criteria for appropriateness of laboratory testing as well as explicit reference to previous literature and/or published guidelines, (ii) the criteria were based on a population that was independent of the study, and (iii) they implemented these criteria in an audit. Studies were excluded if they (i) covered only radiographic imaging or anatomic/surgical pathology testing, (ii) covered only laboratory quality control issues but not the appropriateness of testing, or (iii) had no version available completely in English. To reduce bias, two investigators [J.T. and R.A.] independently evaluated each included study for the validity and appropriateness criteria used in the study, with disagreements resolved by discussion. See File S1 for details on validity, appropriateness, and language criteria. For completeness, we further evaluated all cited literature (manually) and citing literature (using Thomson ISI's Web of Science; Thomson Reuters, NY) for all studies that met selection criteria. For studies identified in this way, we again evaluated all citing and cited literature to identify additional studies. We repeated this step iteratively until no additional studies were found that met selection criteria (Fig. 1).

### Data Extraction and Quality Assessment

For each included study, two investigators [M.Z. and R.A.] abstracted the year of publication, country, and name (s) of test (s) studied. We annotated tests as chemistry, hematology, microbiology, or molecular. We also abstracted (where available) or calculated (where not) the number of tests ordered but not indicated (overutilization) or indicated but not ordered (underutilization) according to the study's criteria; the total number of tests ordered; and the percent overutilization (the number of tests ordered but not indicated divided by the total number of tests ordered ×100) or underutilization (the number indicated but not ordered divided by the total number indicated ×100). Where a study reported results from multiple tests, all tests were considered for inclusion, with each test having its own measurement where possible (its own percent over- or underutilization and its own total number of tests ordered). Where rate (or numerator and/or denominator) or criteria were not available for a test from a given study, that test was excluded. In studies of the pre- vs. post-intervention type, only pre-intervention data were considered, since pre-intervention rates of utilization more likely reflect the landscape of appropriateness across study sites, while post-intervention rates apply only to the site at which the intervention was carried out.

For measures of overutilization, we determined whether the inappropriateness criteria were objective or subjective; restrictive, permissive, or both; and whether they involved initial testing, repeat testing, or both. Test volume (low, medium, or high) was determined as described in the File S1.

### Data Synthesis and Analysis

The percent inappropriate testing (dichotomous data) described different tests and clinical scenarios. Therefore, within the set of study measures for overutilization, and separately for underutilization, we combined study measures using random-effects models and performed meta-regressions. Over- and underutilization data were not combined, since they are calculated as percentages of different denominators (total tests ordered vs. total tests appropriate). Mean rates (proportions) and 95% confidence intervals (CIs), binomial variance, and R-squared (*R* ^2^) were calculated for all groups and subgroups, both separately and controlling for each other (see File S1). We followed the standard practice of adjusting numerators of zero to 0.5 to allow inclusion. We performed subgroup analysis on study measures of over- and underutilization, although the number of study measures of underutilization was too small to draw conclusions about subgroups. Sensitivity analyses were performed to exclude bias due to potential outliers by removing extreme-value studies as appropriate and repeating regressions. We also tested for trends over time (see File S1). All statistical analyses were performed using Stata (version 11.2; StataCorp LP, College Station, Texas, USA) and Microsoft Excel for Mac 2011 (version 14.1.4, Microsoft Corp., Redmond, Washington, USA).

## Results

### Literature search

Our initial literature search identified 34,009 citations. A two-investigator independent manual review yielded 493 studies that were potentially relevant. Applying selection criteria, we excluded 452 because inappropriateness was not defined or calculated (the most common reason for exclusion); the study was a review, survey, case study, or commentary; data were inconsistent; the study was not available in English; and/or the study was a duplicate of another study in the set. Most of the studies for which inappropriateness was not calculated studied the number of tests ordered (total utilization) but not the appropriateness of the orders; the rest reported the number of patients, patient encounters, or providers who encountered or experienced inappropriate testing but not the number of tests.

Another four of the 493 studies (0.8%) were not evaluated because they could not be found despite a thorough search by a professional research librarian [J.W.]. These exclusions left 37 studies. We then applied our eligibility criteria to all studies that cited or were cited by these studies, and repeated this step iteratively until no additional studies were discovered. This additional search resulted in five additional studies, for a total of 42 studies (Figure 1). These 42 reflect agreement among the investigators on all but three studies (42/45; 93% agreement rate); after discussion, these three were excluded.

### Study characteristics

Thirty-eight studies investigated overutilization [15], [17]–[20], [22], [23], [25], [26], [28], [36]–[63]; eight investigated underutilization [25], [45], [48], [57], [64]–[67]; four investigated both [25], [45], [48] [57]. Thirty-one studies used objective criteria for measuring appropriateness [17], [18] [22], [23], [26], [28], [37], [38], [40]–[45], [47]–[49], [52], [54]–[59], [61]–[67] while 11 used subjective criteria [15], [19], [20], [25], [36], [39], [46], [50], [51], [53] [60]. Twenty studies investigated more than one test. Ten studies analyzed the same data using multiple criteria, resulting in multiple study measures for the same laboratory test (s) for each of these studies [23] [25], [38], [45], [57], [64]–[67]. Of the 27 studies that investigated overutilization using objective criteria [17], [18], [22], [23], [26], [28], [37], [38], [40]–[45], [47]–[49], [52], [54]–[59] [61]–[63], 12 used repeat testing as part of their criteria [22], [23], [26], [28], [38], [40], [41], [44], [45], [48], [52], [59] and all but two of these investigated repeat testing exclusively [48], [59]; 18 used non-repeat-based criteria [17], [18], [37], [38], [42], [43], [47]–[49], [54]–[59], [61]–[63]. Sixteen studies used only restrictive criteria [15], [18]–[20], [25], [36]–[38], [42], [47]–[49], [51], [53], [63], [67], 19 used only permissive criteria [17], [22], [23], [26], [28], [39], [41], [43], [44], [50], [52], [55], [59], [60], [62], [64]–[66], and seven used both [45], [46], [54], [56]–[58], [61] (see Table S1 in File S1). Begg's test revealed no obvious publication bias (see File S1).

### Study measures and test coverage

The 42 studies contributed 132 study measures of inappropriateness based on a total of 1,605,095 tests ordered, for an average of 38,217 orders/measure and 3.1 measures/study. Only six study measures (involving prostate-specific antigen [PSA], thyroid studies, and pre-operative testing) were considered screening tests.

Study measures covered 46 of the 50 most frequently ordered laboratory tests at the Beth Israel Deaconess Medical Center (see File S1) and essentially all common tests and panels. These included tests of the basic metabolic panel (e.g. sodium, glucose) [18], [22], [23], [25], [28], [39], [50], [57], [60], [62] and complete blood count (hemoglobin, white blood cell count) [18], [22], [25], [39], [50], [57], [60]; common disease monitoring tests (cholesterol, hemoglobin A1c) [45], [50]; tests of cardiac (creatine kinase) [18], [50], liver (transaminases, alkaline phosphatase) [18], [23], [25], [39], [50], [54], [62], [63], [65], thyroid (thyroxine, thyroid stimulating hormone) [64], and kidney function (blood urea nitrogen, creatinine) [18], [22], [23], [25], [39], [50], [57], [60], [62], tests for anemia (iron, ferritin, B12, serum folate) [39], [40], coagulopathy (prothrombin time, partial thromboplastin time [PTT], protein C, protein S, D-dimer) [18], [39], [42], [50], [55], [57], [61], [66], and inflammation and autoimmunity (C-reactive protein, erythrocyte sedimentation rate, autoimmune antibodies, amylase, lipase) [15], [18], [19], [41], [44], [47], [50], [59], [62]; tests for infection (human immunodeficiency virus Western blot, hepatitis B surface antigen, Venereal Disease Research Laboratory syphilis testing, malarial smear, stool and urine microscopy) [17], [22], [25], [52], [54] [58]; tests for monitoring therapeutic drugs (digoxin, anti-epileptics) [20], [22], [36]–[38], [48], [51], [53]; cancer markers (carcinoembryonic antigen) [43], [44], [46], [56]; and molecular tests (HFE gene mutation).[26] In all, there were 104 chemistry (31 studies) [15], [18]–[20], [22], [23], [25], [36], [39], [41], [43]–[46], [48]–[51], [53], [56], [57], [59], [60], [62]–[65], [67] 24 hematology (13 studies) [18], [22], [25], [39], [40], [42], [47], [50], [55], [57], [60], [61], [66] 10 microbiology (six studies) [17], [22], [25], [52], [54], [58]; and three molecular test study measures (one study) [26].

### Overall rates of inappropriate laboratory testing, 1997–2012

**Figure 2:**
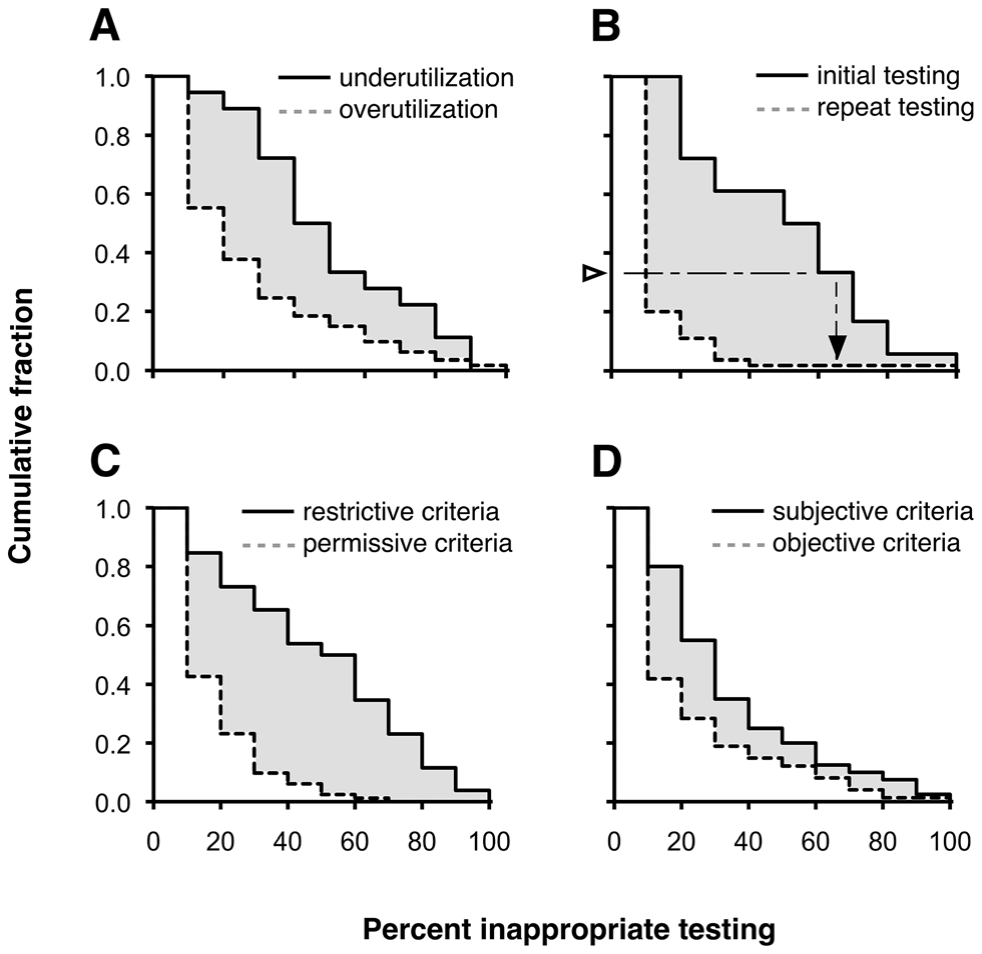
Histograms of Study Measures of Inappropriate Laboratory Test Utilization, 1997–2012. Cumulative distributions of **A**, overutilization vs. underutilization (*P*<0.001); **B**, overutilization, initial vs. repeat testing (*P*<0.001); **C**, overutilization, restrictive vs. permissive criteria (*P*<0.001); and **D**, overutilization, subjective vs. objective criteria (*P* = 0.027). Each curve can be interpreted as the probability (y-axis) that a test was at least as inappropriate as indicated on the x-axis. For example in panel B, a third of study measures of initial testing (open arrowhead, y-axis) found at least 60% inappropriateness (closed arrowhead, x-axis).

The overall mean rate of inappropriate overutilization was 20.6% (95% CI 16.2–24.9%; *n* = 114). The overall mean rate of inappropriate underutilization was higher, at 44.8%, but was based on fewer total study measures (95% CI 33.8–55.8%; *n* = 18; *P* for difference <0.001; Table 1 and Figure 2a).

**Table 1:** Rates of inappropriate laboratory testing, 1997-2012.

Characteristic	Error rate (95% CI)	Difference (95% CI)	n	*S*tratum differences	Variability explained
**Subgroup**					
Overutilization	20.6 (16.2, 24.9)	(reference)	114	*P*<0.001	11%
Underutilization	44.8 (33.8, 55.8)	24.2 (12.5, 36.0)	18		
**Overutilization**					
Initial testing	43.9 (35.4, 52.5)	(reference)	18	*P*<0.001	38%
Repeat testing	7.4 (2.5, 12.3)	−36.5 (−46.4, −26.7)	55		
Both	28.0 (22.2, 33.8)	−15.9 (−5.6, −26.3)	41		
Restrictive criteria	44.2 (36.8, 51.6)	(reference)	26	*P*<0.001	36%
Permissive criteria	12.0 (8.0, 16.0)	−32.2 (−40.6, −23.8)	82		
Subjective criteria	29.0 (21.9, 36.1)	(reference)	40	*P* = 0.004	6%
Objective criteria	16.1 (11.0, 21.2)	−12.9 (−21.6, 4.1)	74		
Low volume	32.2 (25.0, 39.4)	(reference)	36	*P*<0.001	11%
Medium volume	19.8 (12.2, 27.5)	−12.4 (−22.9, −1.8)	31		
High volume	10.2 (2.6, 17.7)	−22.0 (−32.5, −11.6)	32		
Chemistry tests	19.1 (14.3, 24.0)	(reference)	86	NA^a^	2%
Hematology tests	33.3 (20.2, 46.3)	14.1 (0.1, 28.1)	12		
Microbiology tests	23.1 (6.1, 40.2)	4.0 (−13.7, 21.7)	7		
Molecular tests	1.5 (0, 27.4)	−17.6 (−44.0, 8.8)	3		
United States	25.0 (14.0, 36.1)	(reference)	17	*P* = 0.38	<1%
Non-US	19.7 (15.1, 24.4)	−5.3 (−17.3, 6.7)	97		

Subgroup Summary Statistics.

a *P* for stratum differences is meaningful only when there is a natural ordering for categories. *P* for difference of means: chemistry vs. hematology, *P* = 0.05; chemistry vs. microbiology, *P* = 0.65; chemistry vs. molecular, *P* = 0.17; hematology vs. microbiology, *P* = 0.43; hematology vs. molecular, *P* = 0.07; microbiology vs. molecular, *P* = 0.19.

### Overutilization: initial vs. repeat testing

Among study measures of overutilization, the mean rate of inappropriate initial testing – for example, ordering PTT to dose warfarin or low molecular-weight heparin [55] – was 43.9% (95% CI 35.4–52.5%; *n* = 18). The mean rate of inappropriate repeat testing – for example a fourth daily set of serum electrolytes when results from the previous three were all within the reference interval [23] – was 7.4% (95% CI 2.5–12.3%; *n* = 55), a six-fold difference (Table 1 and Figure 2b; *P* for stratum difference <0.001). Forty-one study measures included both initial and repeat-testing criteria. The mean rate for these (28.0%; 95% CI 22.2–33.8%) fell in between rates for initial and repeat testing.

### Overutilization: effect of criteria type

The mean rate for study measures that used restrictive criteria (44.2%; 95% CI 36.8–51.6%; *n* = 26) was five times the rate for those that used permissive criteria (e.g. repeat PSAs within 12 weeks of each other [44]; 12.0%; 95% CI 8.0–16.0%; *n* = 82; *P*<0.001; Table 1 and Figure 2c). In addition, the mean rate for study measures that used subjective criteria (29.0%; 95% CI 21.9–36.1%; *n* = 40) was significantly higher than for those that used objective criteria (16.1%; 95% CI 11.0–21.2%; *n* = 74; *P* = 0.004; Table 1 and Figure 2d). The difference between restrictive and permissive criteria was still significant when controlling for whether study measures were objective vs. subjective (*P*<0.001) and vice versa (*P* = 0.045), and also when controlling for inappropriate initial vs. repeat testing (*P*<0.001) and vice versa (*P*<0.001).

### Overutilization: effect of test volume

The mean rate of inappropriate testing for low-volume tests (e.g. carbamazepine levels, carcinoembryonic antigen; 32.2%; 95% CI 25.0–39.4%; *n* = 36) was significantly higher than for medium- (e.g. albumin, alkaline phosphatase; 19.8%; 95% CI 12.2–27.5%; *n* = 31) or high-volume tests (e.g. those of the basic metabolic panel; 10.2%; 95% CI 2.6–17.7%; *n* = 32; *P*<0.001; Table 1). Although several study measures of repeat testing were of high-volume tests [23], [28], the difference between initial and repeat testing was still significant when controlling for test volume (*P*<0.001) and vice versa (*P*<0.001).

### Overutilization: overall variability

Despite the diversity of tests and clinical settings, over half of the overall variability in overutilization was explained by just three factors: timing (initial vs. repeat testing), type of criteria, and test volume (cumulative *R* ^2^ = 54%).

### Overutilization and underutilization: trends over time

We found no meaningful statistically significant changes in mean rates of inappropriate over- or underutilization between 1997 and 2012 that were robust to sensitivity analysis (see Figure S1 in File S1).

## Discussion

Overuse, underuse, and misuse of health-care resources is estimated at 30 percent [34], [68], [69]. Here we present systematic evidence that laboratory testing is no exception and describe the complex landscape of errors in this highest-volume medical activity.

### Underutilization vs. overutilization

On average, the available evidence suggests that underutilization is more prevalent than overutilization (44.8% vs. 20.6%). This was despite there being only one-fifth the number of studies on underutilization as overutilization during the study period. We do not think the relative lack of studies of underutilization reflects bias in our search methodology, which evaluated over 34,000 studies and succeeded in identifying studies from the previous systematic review [27]. Instead we think it reflects a general emphasis on overutilization relative to underutilization across health care during our study period [34], [68], [69], despite the potential causal relationship between overutilization and upstream underutilization. The relatively small number of study measures of underutilization precluded subgroup analysis and suggests cautious interpretation. However, with a lower 95% confidence bound of 33.8% average underutilization, our results suggest that underutilization in laboratory testing may be a sizeable, underappreciated, and understudied problem that merits further research.

### Initial vs. repeat overutilization

“Inappropriate” and “overutilization” are sometimes used narrowly as synonyms for inappropriate repeat testing – for example, repeat daily electrolytes on inpatients regardless of clinical status (2.5–5.7% inappropriate in one study) [23]. However, our analysis shows that on average, initial testing has a much higher rate of overutilization (43.9% vs. 7.4%) – for example ordering D-dimer despite high pre-test probability for pulmonary embolism (62% of D-dimer orders) [42]. This distinction is robust and remains statistically significant even after controlling for potentially confounding variables such as test volume. There are documented methods for changing test-ordering behavior. These include health information technology-based CDS as well as educational interventions. Our results support focusing such methods on improving initial test ordering.

### Test volume

We find that low-volume tests are ordered inappropriately at a higher rate than medium- or high-volume tests. This may result from a relative lack of familiarity with low- vs. high-volume tests among physicians. However, when taking into account the total number of inappropriate tests, on a per-order (as opposed to per-analyte) basis, high-volume tests likely represent the bigger target for improvement.

### Permissive vs. restrictive criteria

Ideally no medical decision is made without a reason. Decisions to perform surgery, order imaging, or prescribe medication are considered inappropriate absent specific indications. This “guilty-until-proven-innocent” approach to decision-making is exercising what we call restrictive criteria. The opposite, “innocent until proven guilty,” reflects a less skeptical, “why-not?” attitude in which decisions are considered appropriate absent specific contraindications. This is decision-making according to permissive criteria. Our results support the view that decision-making in laboratory testing is too often permissive. By definition, permissive criteria underestimate overall inappropriateness. Restrictive criteria are more explicit about indications and therefore more thorough. For this reason we believe restrictive criteria provide the better measure of inappropriate ordering. Study measures based on restrictive criteria show a mean rate of 44.2% inappropriate overutilization, significantly higher than the mean rate for permissive criteria (12.0%) even after controlling for the fact that inappropriate repeat testing is an example of permissive criteria (because the test is considered appropriate unless inappropriately repeated). For a clearer picture of overutilization, future studies should favor restrictive criteria.

### Subjective vs. objective criteria

Generally, studies that use objective criteria are considered more dependable than those that use subjective criteria. However, that the mean rate of inappropriate overutilization for studies that used subjective criteria (29.0%) was nearly double that for objective criteria (16.1%) merits explanation. It is possible that this difference reflects investigator bias: investigators subjectively believe inappropriate testing is more widespread than it objectively is. However, we favor an alternative explanation: objective criteria for laboratory testing are generally incomplete, while subjective criteria – expert review – judge cases against additional rules and clinical nuances that are often missing from the objective criteria used. We believe the way forward is not to abandon objective criteria but to make objective criteria more complete by working toward defining comprehensive sets of objective indications for the appropriate use of each laboratory test, even as these indications evolve over time.

### Limitations

One could argue that despite an in-depth literature search and coverage of 46 of the 50 most common tests, the total number of studies and study measures reviewed here is small relative to the total number of tests and clinical scenarios encountered in clinical practice. In part this arises from our decision to include studies of utilization only if they explicitly addressed the appropriateness of the tests. This restriction was necessary to avoid confusing the number of tests being ordered (utilization) with the appropriateness of those tests (e.g. to distinguish low overutilization from high underutilization). In part it is simply because tests and scenarios outnumber studies published during the study period. Unfortunately, this imbalance precludes subgroup analysis of underutilization, analysis of the appropriateness of specific tests over time or according to competing guidelines, or differences between inpatient and outpatient settings, small and large hospitals, implicit review and pure subjective review, trainees and experienced practitioners, and generalists and specialists. These are topics for future study. One could also ask whether pooling study measures is desirable given the heterogeneity of tests and testing indications. However, the limited pooling we performed for our subgroup analyses revealed broad, consistent, clinically valuable patterns in laboratory overutilization, and these patterns remained statistically robust when controlling for potential biases of pooling. The availability of more data will allow other techniques and further insights.

One could argue that we cannot completely exclude potential publication bias or selective reporting of results by the studies we included. Indeed, in many of these studies, investigators reported a suspicion of inappropriate testing as part of their motivation. Consequently, we cannot say for sure whether the studies covered in our analysis document the worst offenders or the tip of an “iceberg of errors” [12]. However, we note rough agreement (for overutilization) among rates of inappropriate initial testing, inappropriate testing according to restrictive criteria, and inappropriate testing according to subjective criteria, subgroups with no or relatively little pairwise overlap. Also, Begg's test showed no obvious bias. These observations suggest that our results are likely representative of practice across medicine.

Finally, one could ask how well “appropriateness” approximates the best possible care. All rules have exceptions. Inevitably in medicine situations will arise in which the best decision will seem to run counter to available appropriateness criteria. Also, different appropriateness criteria may contradict each other. Thus, at least by conventional measures, zero inappropriateness is an unrealistic, probably undesirable goal. The included studies do not allow for quantitative definitions of what a realistic or desirable goal might be. However, our review of their appropriateness criteria supports the conclusion that over- and underutilization are both more common than they should be.

### Conclusions

Inappropriate testing is not just unnecessary repeat blood draws. Our work reveals a landscape of inappropriate testing where rates vary systematically according to setting, test volume, and criteria in ways that can inform clinical practice and future research. For example, focusing on ordering the right test during initial evaluation, as opposed to reducing repeat testing, may have the greater impact on reducing errors and improving care.

What about reducing cost? Laboratory testing itself accounts for only a tiny fraction (∼3–5%) of healthcare spending [2]. The true costs associated with testing include the costs or savings of the downstream activities that testing leads to or prevents. The costs of these downstream activities – prescriptions, imaging, surgeries, hospital stays – dwarf the cost laboratory testing [2], [70]. Economic models of how testing influences these activities would be useful. Meanwhile, insofar as testing is considered appropriate only if it supports the standard of care, which in turn is defined according to patient outcomes, improving laboratory utilization should lead to more cost-effective care, regardless of whether more appropriate utilization leads to fractionally lower, or even fractionally higher, testing costs. We suggest further study of over- and underutilization in tandem, and in the context of downstream costs and outcomes, to learn how best to improve the efficiency and effectiveness of care.

## Supporting Information

Checklist S1 **PRISMA Checklist.**
(PDF)

File S1 1. Search Methodology
1–1. Search notes
1–1–1.Note regarding date ranges
1–1–2.Note regarding English language requirement
1–1–3. Additional assessment of validity: test for sensitivity
1–1–4. Test for publication bias
1–2. Detailed review criteria
1–3. Assessment of testing volume
1–4. Search details
1–4–1. Medline search details
1–4–2. Embase search details
1–4–3. BIOSIS search details
1–4–4. CINAHL search details
1–4–5.Cochrane database search details
1–5. **Checklist S2, MOOSE checklist.**
2. Additional Results
3.Supporting Information Tables and Figures.
**Figure S1, Overutilization and Underutilization over Time, 1997–2012.**
**Table S1, Study Measures of Inappropriate Testing, 1997–2012.** **Table S2, Overutilization and Underutilization over Time: Representative Analyses.**
4. References.
(DOCX)
