# Supplementary material for: The Landscape of Inappropriate Laboratory Testing: A 15-Year Meta-Analysis
Source: PLoS One. 2013 Nov 15;8(11):e78962. doi: 10.1371/journal.pone.0078962 (PMC3829815; doi:10.1371/journal.pone.0078962)
Supplement: File S1 — 1. Search Methodology 1–1. Search notes 1–1–1.Note regarding date ranges 1–1–2.Note regarding English language requirement 1–1–3. Additional assessment of validity: test for sensitivity 1–1–4. Test for publication bias 1–2. Detailed review criteria 1–3. Assessment of testing volume 1–4. Search details 1–4–1. Medline search details 1–4–2. Embase search details 1–4–3. BIOSIS search details 1–4–4. CINAHL search details 1–4–5.Cochrane database search details 1–5. Checklist S2, MOOSE checklist. 2. Additional Results 3.Supporting Information Tables and Figures. Figure S1, Overutilization and Underutilization over Time, 1997–2012. Table S1, Study Measures of Inappropriate Testing, 1997–2012. Table S2, Overutilization and Underutilization over Time: Representative Analyses. 4. References. (DOCX) [file pone.0078962.s002.docx]

**Supporting Information**

This Supporting Information contain the following sections and subsections:

S1. Search Methodology

S1-1. Search notes

S1-1-1. Note regarding date ranges

S1-1-2. Note regarding English language requirement
 S1-1-3. Additional assessment of validity: test for sensitivity

S1-1-4. Test for publication bias

S1-2. Detailed review criteria

S1-3. Assessment of testing volume

S1-4. Search details

S1-4-1. Medline search details
 S1-4-2. Embase search details
 S1-4-3. BIOSIS search details
 S1-4-4. CINAHL search details
 S1-4-5. Cochrane database search details

S1-5. MOOSE checklists

S2. Additional Results

S3. Supporting Information Tables and Figures

Supporting Information Figure 1. Overutilization and Underutilization over Time, 1997-2012

Supporting Information Table 1. Study Measures of Inappropriate Testing, 1997-2012

Supporting Information Table 2. Overutilization and Underutilization over Time: Representative Analyses

S4. References

**S1. Search Methodology**

***S1-1. Search notes***

*S1-1-1. Note regarding date ranges*

Date ranges for BIOSIS, CINAHL, and Cochrane database searches can be set only by year, but not by month or day. Thus, 1997 was included in the year range, and studies from January to September 1997 were removed manually.

*S1-1-2. Note regarding English language requirement*

The initial literature search included studies published in all languages to provide a global context to our results. Of 24,727 unique Medline results,^[[1]](#footnote-1)^ a total of 2,617 studies (11%) were in a language other than English. We reviewed the title and abstract for each of these results, which Medline provides in English. Of the 2,617, fewer than 1% were selected for in-depth review.

For in-depth review, we required the language to be in English, as this is the primary language of the investigators. As described above, our initial screen enriched for English-language studies, and the number of non-English-language studies included as a result of initial review was small. Thus, we did not expect a subsequent language requirement to bias the global context of our study. Nevertheless, we performed a detailed analysis of the 24,727 unique Medline results by asking what fraction of studies from non-English-speaking countries were nevertheless in English. We counted the number of studies in English and the number not in English for studies from the US and Canada (North American English-speaking countries), for the UK and Australia (major non-North American English-speaking countries), and for all other countries in the world. As expected, we found that 13,085/15,695=83.4% of non-US studies were in English and 9,838/12,440=79.1% of studies produced outside of the United States, Canada, United Kingdom, or Australia were likewise in English. Thus, limiting to English would impose at most a 21% potential bias on initial results from non-English-speaking countries had we reviewed titles and abstracts from only English-language studies; and, as only about 5% of studies selected for in-depth review were not in English, the actual potential bias is at most about 5%. Finally, there is no reason to think that non-English-language studies are more likely to meet inclusion criteria than English-language studies. The overall rate of studies that met inclusion criteria was under 10% (36/489^[[2]](#footnote-2)^ studies). In practical terms, the bias due to English-language restriction is more likely on the order of 10% times 5%, or 0.5%.

*S1-1-3. Additional assessment of validity: test for sensitivity*

To further assess the validity of this literature search strategy, we applied it to the period before 1997, covered by a previous study ([1](#_ENREF_1)), and counted the number of studies that our search returned that were also returned in the that review. Doing so identified 27 of the studies included in a previous systematic review ([1](#_ENREF_1)). In contrast, the systematic component of the previous review’s Medline search identified only 12 studies; the authors of that review discovered the remainder by hand. Thus, measured against the only previous study of this kind, the literature search method used in the present work was more comprehensive.

*S1-1-4. Test for publication bias*

Historically, funnel plots provided a means for evaluating publication bias. However, these plots are known to be prone to subjective interpretation ([2](#_ENREF_2)). Therefore we used Begg’s test to test for a correlation between standardized effect sizes and their variances in our study measures of overutilization and underutilization. This showed no evidence of publication bias (*P*=0.44 and *P*=0.20, respectively). Egger’s test complements Begg’s test by focusing on the intercept of the regression between effect size and variance (or measures thereof). A positive intercept indicates that smaller studies have a larger effect than larger studies (resulting in skewness of the funnel graph). However, in our study, we actually expected a positive intercept, and therefore significant bias by Egger’s test, since our smaller studies are often low-volume studies, and we find that low-volume studies have larger effect sizes (which we attribute to physicians’ relative lack of familiarity with low- vs. high-volume tests). (Note if there were a conflation based on bias, we would expect high variance of low-volume studies and therefore no significant difference in mean from high-volume studies, whereas we do find a significant difference; see Table 1 and discuss in the Discussion.)

***S1-2. Detailed review criteria***

To assess validity of appropriateness criteria (eligibility criterion 1 in the main text), two investigators [R.A. and J.T.] independently evaluated each included study for five requirements:

(i) The study was required to have a clear definition of the appropriateness or inappropriateness criteria; either based on expert review (subjective) or defined objective criteria. These criteria must be used to evaluate a defined study population. For example, one study ([3](#_ENREF_3)) was rejected due to lack of explicitly listed guidelines: the study lists its inappropriateness criteria as “noncompliance with hospital guidelines” but does not explicitly state the guidelines in the study, or provide a link or reference to the guidelines.

(ii) The study criteria were required to be consistent with regional, national or international guidelines at the time it was performed. If not supported by explicitly referenced guidelines, the criteria were required to not contradict established guidelines at the time it was performed.

(iii) The study criteria were required to be justified by previously published literature that was referenced and available for review. For example, one study ([4](#_ENREF_4)) was rejected because it lacked supporting literature for the times used to define inappropriate early redundant repeat testing.

(iv) The study was required to have clinical plausibility based on justifications made. For example, one study ([5](#_ENREF_5)) was accepted based on justification that genetic polymorphism testing should not change and only rarely requires duplicate testing for confirmation; another ([6](#_ENREF_6)) was accepted based on the justification that partial thromboplastin monitoring for warfarin or low molecular weight heparin is inappropriate and prothrombin monitoring of unfractionated heparin or low molecular weight heparin is inappropriate.

(v) The study was required to have clearly reported data for each individual test being evaluated including total number of tests and total number of appropriate or inappropriate tests or rate of appropriate or inappropriate tests. The data reported must be consistent throughout the study. Individual tests not meeting this criterion were excluded from our analysis. However, other individual tests in the same study were considered for inclusion if they satisfied the other criteria. For example, one study reported individual test results in a table with multiple tests meeting the above criteria, which were included in the analysis ([7](#_ENREF_7)). The table also had multiple other tests consolidated into an “other” category. Given we could not determine total number of tests or number of appropriate or inappropriate tests for the individual tests in the “other” category, those tests were excluded from the analysis.

Two investigators [J.T. and R.A.] independently evaluated each identified study for bias. Studies that were deemed to have significant bias were discussed for exclusion from our analysis. As mentioned in the Methods of the main text, in order to decrease bias, post-intervention data (in which an intervention was undertaken to increase appropriate testing or decrease inappropriate testing) was excluded from our analysis.

***S1-3. Assessment of testing volume***

The Beth Israel Deaconess Medical Center (BIDMC) is a 585-bed tertiary care center in Boston, MA. Its clinical laboratories provide a wide range of laboratory testing services for both inpatient and outpatient care. As a metric of the clinical coverage of the tests in the included studies, we ranked all tests performed at BIDMC in 2010 by volume and counted the ones that appeared in the studies included for review. We segmented tests into low (<100,000 tests/year), medium (100,000-1 million tests/year), and high volume (>1 million tests/year) categories, where data for those tests was available from BIDMC.

***S1-4. Search details***

*S1-4-1. Medline search details*

Medline was searched with date ranges of October 1, 1997 to January 1, 2012 using the following query (which included subheadings):

"Laboratory Techniques and Procedures/statistics and numerical data"[Mesh] OR "Laboratory Techniques and Procedures/utilization"[Mesh] OR “Laboratory Techniques and Procedures/economics”[Mesh] OR "Laboratories/statistics and numerical data"[Mesh] OR "Laboratories/utilization"[Mesh] OR “Laboratories/economics”[Mesh] OR "Unnecessary Procedures/statistics and numerical data"[Mesh] OR "Unnecessary Procedures/utilization"[Mesh] OR “Unnecessary Procedures/economics”[Mesh] OR "Diagnostic Techniques and Procedures/statistics and numerical data"[Mesh] OR "Diagnostic Techniques and Procedures/utilization"[Mesh] OR “Diagnostic Techniques and Procedures/utilization”[Mesh]

AND

utilization[tiab] OR overutilization[tiab] OR "laboratory tests"[tiab] OR "laboratory test"[tiab] OR optimal[tiab] OR inappropriate[tiab] OR appropriate[tiab] OR repeat[tiab] OR unnecessary[tiab] OR necessary[tiab] OR reliability[tiab] OR reliable[tiab] OR guideline[tiab] OR contraindicated[tiab] OR duplication[tiab] OR efficiency[tiab] OR "clinical audits"[tiab] OR "clinical audit"[tiab] OR "Utilization Review"[Mesh] OR "Guideline Adherence"[Mesh] OR "Practice Guidelines as Topic"[Mesh] OR "Peer Review, Health Care"[Mesh] OR "Physician's Practice Patterns"[Mesh] OR "Quality Assurance, Health Care"[Mesh].

The query was repeated without subheadings:

unnecessary[tiab] OR duplication[tiab] OR efficiency[tiab] OR inappropriate[tiab] OR overutilization[tiab] OR underutilization[tiab] OR "quality control"[tiab] OR "quality assurance"[tiab] OR guidelines[tiab] OR utilization[tiab] OR "utilization review"[tiab] OR "clinical audits"[tiab] OR "clinical audit"[tiab] OR "Attitude of Health Personnel"[Mesh] OR "Physician's Practice Patterns"[Mesh] OR "Guidelines as Topic"[Mesh] OR "Peer Review, Health care"[mesh] OR "guideline adherence"[mesh] OR "Utilization Review"[Mesh] OR "Quality Assurance, Health Care"[Mesh] OR "Health Services Misuse"[mesh] OR "optimal"[tiab] OR "inappropriate"[tiab] OR "appropriate"[tiab] OR "repeat"[tiab] AND ("Laboratories, Hospital"[Mesh] OR "Laboratory Techniques and Procedures"[Mesh] OR (("Diagnostic Services"[Mesh] OR "Diagnostic techniques and procedures"[mesh]) AND ("laboratory"[tiab] OR "laboratories"[tiab])) AND laboratory[tiab] OR "laboratory utilization"[tiab] OR "Clinical Chemistry Tests"[mesh] OR "laboratory tests"[tiab] OR "laboratory test"[tiab]).

*S1-4-2. Embase search details*

Embase was searched using the following query:

['clinical laboratory'/exp/mj OR 'laboratory diagnosis'/exp/mj AND 'hospital laboratory'/exp/mj OR 'diagnostic test'/exp AND ('utilization review'/exp OR 'health economics'/exp) AND [embase]/lim NOT [medline]/lim AND [1998-2011]/py.]

*S1-4-3. BIOSIS search details*

BIOSIS was searched for meeting abstracts using the following query:

TI=(utilization OR audit OR unnecessary) AND TI=(laboratory OR laboratories)
Refined by: Document Type=( MEETING )
Timespan=1997-2012. Databases=BIOSIS Previews.
Lemmatization=On

*S1-4-4. CINAHL search details*

CINAHL was searched using the following query:

S2 (MH "Diagnosis, Laboratory+") 77729

S3 (MH "Utilization Review") OR TX utilization 107919
S4 (MH "Unnecessary Procedures") 640
S5 MH "Clinical Laboratories+" 84
S6 S2 or S4 or S5
S7 (MH "Guideline Adherence") 2021
S8 (MH "Quality Assurance+") 68288
S9 (MH "Practice Patterns") 3029
S10 (MH "Quality Assessment+") 44421
S11 S3 or S7 or S8 or S9 or S10 169022
S12 "clinical audit" 550
S13 TX duplication OR TX efficiency OR TX inappropriate OR TX reliable OR TX repeat 130099
S14 S11 or S13 4290
# Query Limiters/Expanders Last Run Via Results S15 S6 and S14
Limiters - Published Date from: 19971001-20120101; Human

*S1-4-5. Cochrane database search details*

The Cochrane database was searched using the following query, with date range of 1997-2012:

Laboratory AND clinical AND utilization

***S1-5. MOOSE Checklist***

| **Criteria** | | **Brief description of how the criteria were handled in the meta-analysis** |
| --- | --- | --- |
| **Reporting of background should include** | |  |
| √ | Problem definition | Inappropriate laboratory testing is thought to be widespread in clinical practice, but its overall prevalence is unknown. |
| √ | Hypothesis statement | Inappropriate laboratory utilization can be quantified via systematic review of literature. Analyses of clinically relevant subcategories—overutilization vs. underutilization, subjective vs. objective criteria, “permissive” vs. “restrictive” criteria, testing volume, initial vs. repeat testing, publication year, by type (chemistry, hematology, microbiology, molecular) and geography—in laboratory utilization can reveal meaningful heterogeneities in testing appropriateness. |
| √ | Description of study outcomes | Rates of inappropriate laboratory over- and underutilization across clinical medicine were determined and compared for each of the subcategories above. |
| √ | Type of exposure or intervention used | NA |
| √ | Type of study designs used | We included prospective and retrospective studies with specified valid criteria for appropriateness of laboratory testing on human subjects. |
| √ | Study population | No geographic restrictions were made. The study was required to be available completely in English. |
| **Reporting of search strategy should include** | |  |
| √ | Qualifications of searchers | MZ is a medical student at Harvard Medical School studying utilization and evidence-based medicine. ED is a published epidemiologist who teaches meta-analysis at the Harvard School of Public Health. JT is a hospitalist at Beth Israel Deaconess Medical Center. JW is a research librarian at Harvard Medical School’s Countway Library of Medicine. RA is a practicing clinical pathologist and clinical laboratory director at the Beth Israel Deaconess Medical Center and Harvard Medical School. |
| √ | Search strategy, including time period included in the synthesis and keywords | Medline, Embase, BIOSIS,CINAHL, and Cochrane databases were searched from October 1, 1997 to January 1, 2012 with keywords and details as described. Thomson ISI’s Web of Science was used to evaluate citing literature. |
| √ | Databases and registries searched | Medline, Embase, BIOSIS, CINAHL, Cochrane, and Thomson ISI’s Web of Science. |
| √ | Search software used, name and version, including special features | Medline was searched using PubMed. The remaining databases above each have their own search interfaces (see details elsewhere in the Supplementary Appendix). EndNote X4 (Thomson Reuters) was used for citation-handling. |
| √ | Use of hand searching | We hand-searched bibliographies of included studies. |
| √ | List of citations located and those excluded, including justifications | Included citations are provided in the bibliography/references of the manuscript. Details of the literature search process are outlined in Fig. 1 of the main text. Complete citations available upon request. |
| √ | Method of addressing articles published in languages other than English | We reviewed titles and abstracts irrespective of language. We excluded studies for which a version was not available entirely in English. |
| √ | Method of handling abstracts and unpublished studies | Abstracts and unpublished studies were excluded. Meeting notes found during the search process were also excluded. |
| √ | Description of any contact with authors | None. |
| **Reporting of methods should include** | |  |
| √ | Description of relevance or appropriateness of studies assembled for assessing the hypothesis to be tested | For each citation, two investigators independently screened the title and abstract for potential relevance and the results were combined. For citations considered potentially relevant, the study was evaluated in depth according to the following inclusion and exclusion criteria: studies were included if (1) they specified valid criteria for appropriateness of laboratory testing as well as explicit reference to previous literature and/or published guidelines; (2) the criteria were determined based on a population that was independent of the study; and (3) they implemented these criteria in an audit. Studies were excluded if they (1) covered only radiographic imaging or anatomic/surgical pathology testing, (2) covered only laboratory quality control issues but not the appropriateness of testing, or (3) had no version available completely in English. To reduce bias, two investigators independently evaluated each study for validity and appropriateness, with disagreements resolved by discussion. |
| √ | Rationale for the selection and coding of data | Data was selected and coded to allow investigation of the above-listed hypotheses. Therefore the following data was selected and coded as dichotomous data: year of publication, country, tests studied, test type (service or category), whether inappropriate utilization constituted over- or underutilization, whether it involved initial or repeat testing, whether inappropriateness criteria was objective or subjective and permissive or restrictive. |
| √ | Assessment of confounding | Stratification of overall rates of overutilization according to initial vs. testing, restrictive vs. permissive criteria, subjective vs. objective criteria, testing volume (low, medium, high), test type (chemistry, hematology, microbiology, molecular) and geography (United States vs. non-US). Effects for each subcategory were controlled for confounding by other covariates. |
| √ | Assessment of study quality, including blinding of quality assessors; stratification or regression on possible predictors of study results | For study quality, two investigators independently (blinded) assessed identified studies for validity and appropriateness with disagreements resolved by discussion. The two investigators had an agreement rate of 93% (42/45 studies). |
| √ | Assessment of heterogeneity | Study measures were analyzed using random-effects models and meta-regressions. An R^2^ analysis was performed, showing that our subgroups explain nearly 60% of the heterogeneity in rates of inappropriate utilization. Analysis of between-group heterogeneity was the purpose of the subgroup analysis. This exploration of between-study heterogeneity uncovered heterogeneity in permissive vs. restrictive, objective vs. subjective, high vs. low volume, and initial vs. repeat testing. |
| √ | Description of statistical methods in sufficient detail to be replicated | We meta-analytically pooled results via random effects models and meta-regression analyses to assess temporal trends and effect modification by covariates. We performed sensitivity analyses by repeating analyses to test the affect of large-n and extreme-value measures. We binned study measures manually by year and performed sensitivity analyses by manually choosing different bins. All statistical analyses were performed using Stata (version 11.2; StataCorp LP, College Station, Texas, USA; *metareg*, *metabias*, *testparm*, *adjust* commands) and Microsoft Excel for Mac 2011 (version 14.1.4, Microsoft Corp., Redmond, Washington, USA). |
| √ | Provision of appropriate tables and graphics | Figures 1 and 2 demonstrate the search methodology, search results, and the distribution of measurements and differences among key subsets. The Supplementary Figure is representative of changes over time. Table 1 summarizes the rates of inappropriate laboratory testing and presents subgroup analyses. |
| **Reporting of results should include** | |  |
| √ | Graph summarizing individual study estimates and overall estimate | Figure 2 presents four histograms that summarize the overall study estimates for over- and underutilization and individual estimates by overutilization subgroup. |
| √ | Table giving descriptive information for each study included | The Supplementary Table presents all study measures. |
| √ | Results of sensitivity testing | We removed extreme-value or high-n measurements and demonstrated that this removal did not change conclusions, as described in the manuscript text. |
| √ | Indication of statistical uncertainty of findings | 95% confidence intervals are presented with all mean-effect calculations. *R*^2^ values and results of sensitivity analyses are as described. |
| **Reporting of discussion should include** | |  |
| √ | Quantitative assessment of bias | Potential publication bias was assessed via Begg’s and Egger’s tests, with sensitivity examined via trim-and-fill method. Confounding by various covariates and the robustness of results across covariates was assessed via stratified analyses of covariates. |
| √ | Justification for exclusion | Exclusions were made according to pre-defined inclusion and exclusion criteria, as described. |
| √ | Assessment of quality of included studies | Two investigators independently assessed studies for quality and validity as described. |
| **Reporting of conclusions should include** | |  |
| √ | Consideration of alternative explanations for observed results | These are commented on in the Discussion section where relevant. |
| √ | Generalization of the conclusions | On average, the rates of overutilization and underutilization of laboratory tests were 21% and 45%, respectively. Overutilization in initial testing is six times that in repeat testing. Overutilization defined by restrictive criteria—testing considered inappropriate absent indications— is three times that by permissive criteria. Overutilization for low-volume tests is three times that for high-volume tests. Overall nearly a billion tests may have been ordered in error in the United States last year. Potentially, another billion tests should have been ordered but were not. |
| √ | Guidelines for future research | We recommend future studies that focus on analyzing over- and underutilization in tandem and in the context of downstream costs and preventable adverse outcomes. |
| √ | Disclosure of funding source | No external funding was used. |

**S2. Additional results**

***Inappropriate overutilization: other subgroup analyses***

We found no significant differences among chemistry, hematology, microbiology, or molecular tests (*P*=0.11).

***Trends over time***

To test for trends in inappropriate ordering over the study period (1997-2012), we tested for a linear trend over time by performing a regression of all data vs. categorical time, and for nonlinear trends via regressions with spline terms by binning data into three bins that contained similar numbers of study measures per interval. Sensitivity analyses were performed by repeating regressions using multiple different date cutoffs for two as well as three bins.

**S3. Supporting Information Tables and Figures**

**Supporting Information Figure 1. Overutilization and Underutilization over Time, 1997-2012**


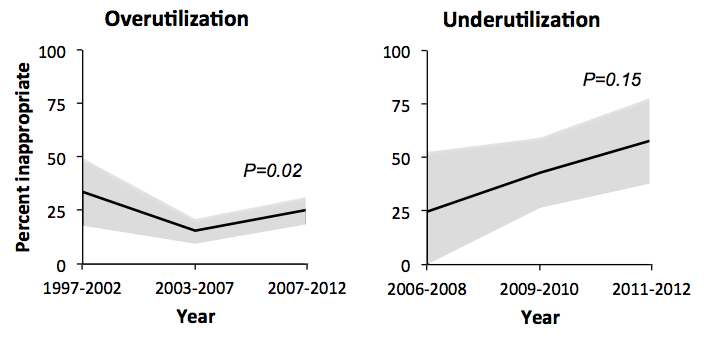


**Figure S1. Overutilization and Underutilization over Time, 1997-2012.** Shown are means (lines) and 95% confidence intervals (shaded bands) for the indicated time periods

**Supporting Information Table 1. Study Measures of Inappropriate Testing, 1997-2012**

| **Test(s)** | **Summary of criteria** | **Count** | **Total** | **%** | **Type** |
| --- | --- | --- | --- | --- | --- |
| Chemistry-20 profile; aminophylline, digoxin, vancomycin, gentamicin, tobramycin, and amikacin levels; urinalysis; urine, stool, sputum culture; *Clostridium difficile*toxin assay; fibrin split products | Repeats within a given time interval (8-20 hours, depending on the test); see Ref. Table 1 ([8](#_ENREF_8)) | 257 | 13,847 | 0.6 | BOYP |
| Digoxin | Inappropriate unless ordered to test for subtherapeutic response (no improvement or worsening of congestive heart failure or atrial fibrillation or flutter; suspected noncompliance; concomitant use of an interacting drug, suspected malabsorption), suspected toxicity (arrythmias suspected to be caused by digoxin; noncardiac signs or symptoms of digoxin toxicity); on a high-risk patient; for initiation of therapy or dosage adjustment after steady-state reached; if no previous level within the past nine months (inpatients only); or for routine annual monitoring (outpatients only); see Ref. Table 1 ([9](#_ENREF_9)) | 251 | 354 | 70.8 | BONR |
| Complete blood count, urea, creatinine, electrolytes, liver function tests, cardiac enzymes, clotting studies, crossmatch, glucose, amylase, arterial blood gas | Inappropriate unless compliant with guidelines for initial emergency room assessment for various common clinical conditions (ischemic chest pain, arrhythmia, heart failure, upper and lower gastrointestinal bleeding; see Ref. Box 1 for complete list) ([10](#_ENREF_10)) | 8,895 | 14,300 | 62.2 | BONR |
| Prostate-specific antigen | Inappropriate if screening of asymptomatic men >75 years old or asymptomatic patients with less than 10 years of life expectancy; screening asymptomatic men <50 years old with no risk factors, or <40 years old with risk factors ([11](#_ENREF_11)) | 87 | 433 | 20.1 | BONT |
| Arterial blood gas | Inappropriate unless compliant with conditions set forth in a comprehensive author-developed guideline (see Ref. Fig. 1) ([12](#_ENREF_12)) | 485 | 1,031 | 47.0 | BONR |
| Digoxin | Inappropriate unless adequate indication (suspected toxicity, newly initiated therapy, dose adjustment in patients with unstable renal function, subtherapeutic response despite adequate dose, high-risk patient, digoxin therapy uncertain or unknown, potential drug interaction, suspected abuse, decision for future therapy), blood sample drawn unless adequate indication (suspected toxicity, newly initiated therapy, dose adjustment in patients with unstable renal function ([13](#_ENREF_13)) | 125 | 210 | 59.5 | SOIR |
| Autoimmune screens (anti-nuclear, mitochondrial, gastric parietal, smooth muscle, reticulin, thyroid antibodies), rheumatoid factor, and immunoglobulin levels | Repeat order inappropriate if within three months of previous order ([14](#_ENREF_14)) | 1,830 | 25,067 | 7.3 | BOYP |
| Autoimmune screens (anti-nuclear, mitochondrial, gastric parietal, smooth muscle, reticulin, thyroid antibodies), rheumatoid factor, and immunoglobulin levels | Repeat order inappropriate if within 2-4 weeks of previous order ([14](#_ENREF_14)) | 577 | 25,067 | 2.3 | BOYP |
| Phenytoin, valproate, carbamazepine | Inappropriate unless adequate indication (newly initiated therapy, insufficient response despite adequate dose, suspected pharmacokinetic change, calculation of individual pharmacokinetics of phenytoin, suspected toxicity, drug-drug interaction, following dose adjustment of phenytoin, ≤6 hours of an epileptic seizure) and correct sampling time (see Ref. Appendix 1) ([15](#_ENREF_15)) | 313 | 602 | 52.0 | SOIR |
| Urea, aspartate aminotransferase, lactate dehydrogenase, amylase, alkaline phosphatase, bilirubin | Inappropriate if ordered for vague upper abdominal complaints in the absence of jaundice or for screening purposes ([16](#_ENREF_16)) | 63 | 478 | 13.2 | BONP |
| Phenytoin, valproic acid, carbamazepine, phenobarbital | Inappropriate unless ordered within six hours after a seizure recurrence, suspicion of dose-related toxicity or noncompliance; otherwise, appropriate only if the blood sample is drawn in steady-state conditions (four half-lives) on an unchanged dose regimen, as a baseline after start of drug, as control after a change, after adding a drug with a potential drug-drug interaction, or after change in liver or GI function (see Ref. Table 1) ([17](#_ENREF_17)) | 61 | 241 | 25.3 | BOYR |
| Phenytoin, valproic acid, carbamazepine, Phenobarbital | Inappropriate unless ordered within six hours after a seizure recurrence, suspicion of dose-related toxicity or noncompliance; otherwise, appropriate only if the blood sample is drawn in steady-state conditions (four half-lives) on an unchanged dose regimen, as a baseline after start of drug, as control after a change, after adding a drug with a potential drug-drug interaction, or after change in liver or GI function (see Ref. Table 1) ([17](#_ENREF_17)) | 43 | 241 | 17.8 | BONR |
| Tumor markers including CA-125, CA15-3, CEA, and AFP | Inappropriate unless ordered in accordance with literature as a baseline, for monitoring, diagnosis, surveillance, or screening; or if three or more orders for the same marker within two months, if CA-125 or 15-3 were ordered in male patients (unless they have metastatic breast cancer), or if a marker was ordered not in relation to a cancer diagnosis; see Ref. Table 1 ([18](#_ENREF_18)) | 148 | 476 | 31.0 | SOIT |
| Hepatitis A serology (IgG and IgM) | Anti-HAV IgM inappropriate for determining immune status; anti-HAV total inappropriate for suspected acute infection (orders of both inappropriate for one or the other of these two situations) ([19](#_ENREF_19)) | 1,472 | 2,944 | 50.0 | BONT |
| Lyme disease serologic testing | Appropriate if patient had oligoarticular arthritis, cranial neuritis, lymphocytic meningitis, atrioventricular block, or carditis; inappropriate if ordered in absence of findings consistent with disseminated Lyme disease, in asymptomatic patients, in presence of clinician-diagnosed erythema migrans, in patients receiving empiric antibiotics, as test for clearance of infection (see Ref. Table 1) ([20](#_ENREF_20)) | 95 | 356 | 26.7 | BONT |
| Phenobarbitone | Inappropriate unless ordered for documented suspicion of poor adherence or toxicity ([21](#_ENREF_21)) | 6 | 7 | 85.7 | SOIR |
| Phenytoin | Inappropriate unless ordered for documented suspicion of poor adherence or toxicity ([21](#_ENREF_21)) | 36 | 50 | 72.0 | SOIR |
| Lamotrigine | Inappropriate unless ordered for documented suspicion of poor adherence or toxicity ([21](#_ENREF_21)) | 3 | 8 | 37.5 | SOIR |
| Carbamazepine | Inappropriate unless ordered for documented suspicion of poor adherence or toxicity ([21](#_ENREF_21)) | 14 | 22 | 63.6 | SOIR |
| Valproate | Inappropriate unless ordered for documented suspicion of poor adherence or toxicity ([21](#_ENREF_21)) | 16 | 27 | 59.3 | SOIR |
| CA15-3 | Inappropriate if order repeated within 12 weeks (see Ref. Table 1) ([22](#_ENREF_22)) | 1,357 | 4,582 | 29.6 | BOYP |
| Alpha-fetal protein | Inappropriate if order repeated within 12 weeks (see Ref. Table 1)([22](#_ENREF_22)) | 5,153 | 24,072 | 21.4 | BOYP |
| Prostate-specific antigen | Inappropriate if order repeated within 12 weeks (see Ref. Table 1) ([22](#_ENREF_22)) | 665 | 3,244 | 20.5 | BOYP |
| IgG, IgA, IgM levels | Inappropriate if order repeated within 4 weeks (see Ref. Table 1) ([22](#_ENREF_22)) | 1,164 | 7,059 | 16.5 | BOYP |
| Carcinoembryonic antigen | In setting of colon cancer, inappropriate if order repeated within 12 weeks (see Ref. Table 1) ([22](#_ENREF_22)) | 1,718 | 10,875 | 15.8 | BOYP |
| Carcinoembryonic antigen | In setting of breast cancer, inappropriate if order repeated within 12 weeks (see Ref. Table 1) ([22](#_ENREF_22)) | 471 | 5,438 | 8.7 | BOYP |
| Anti-nuclear antibodies | Inappropriate if order repeated within four weeks (see Ref. Table 1) ([22](#_ENREF_22)) | 292 | 6,161 | 4.7 | BOYP |
| Rheumatoid factor | Inappropriate if order repeated within 4 weeks (see Ref. Table 1) ([22](#_ENREF_22)) | 156 | 3,843 | 4.1 | BOYP |
| Anti-double-stranded DNA antibodies | Inappropriate if order repeated within four weeks (minimum estimate; compare Ref. Table 2 with 6-12 weeks or 6-12 months in Ref. Table 1) ([22](#_ENREF_22)) | 1,028 | 6,150 | 16.7 | BOYP |
| Anti-smooth-muscle antibodies | Inappropriate if order repeated within four weeks (see Ref. Table 1) ([22](#_ENREF_22)) | 42 | 1,312 | 3.2 | BOYP |
| Anti-extractable nuclear antibodies | Inappropriate if order repeated within four weeks (see Ref. Table 1) ([22](#_ENREF_22)) | 150 | 4,742 | 3.2 | BOYP |
| Anti-mitochondrial antibodies | Inappropriate if order repeated within four weeks (see Ref. Table 1) ([22](#_ENREF_22)) | 37 | 1,427 | 2.6 | BOYP |
| Anti-gastric parietal cell antibodies | Inappropriate if order repeated within four weeks (see Ref. Table 1) ([22](#_ENREF_22)) | 8 | 501 | 1.6 | BOYP |
| Potassium | Repeat was inappropriate when four or more sequential results were all within the reference interval ([23](#_ENREF_23)) | 6,081 | 106,685 | 5.7 | BOYP |
| Potassium | Repeat was inappropriate when two prior sequential results were both within the reference interval ([23](#_ENREF_23)) | 5,548 | 106,685 | 5.2 | BOYP |
| Creatinine | Repeat was inappropriate when four or more sequential results were all within the reference interval ([23](#_ENREF_23)) | 4,915 | 100,298 | 4.9 | BOYP |
| Bicarbonate | Repeat was inappropriate when four or more sequential results were all within the reference interval ([23](#_ENREF_23)) | 4,128 | 84,241 | 4.9 | BOYP |
| Bicarbonate | Repeat was inappropriate when two prior sequential results were both within the reference interval ([23](#_ENREF_23)) | 3,707 | 84,241 | 4.4 | BOYP |
| Creatinine | Repeat was inappropriate when two prior sequential results were both within the reference interval ([23](#_ENREF_23)) | 4,112 | 10,0298 | 4.1 | BOYP |
| Urea | Repeat was inappropriate when four or more sequential results were all within the reference interval ([23](#_ENREF_23)) | 3,952 | 98,808 | 4.0 | BOYP |
| Chloride | Repeat was inappropriate when four or more sequential results were all within the reference interval ([23](#_ENREF_23)) | 3,385 | 94,024 | 3.6 | BOYP |
| Urea | Repeat was inappropriate when two prior sequential results were both within the reference interval ([23](#_ENREF_23)) | 3,458 | 98,808 | 3.5 | BOYP |
| Chloride | Repeat was inappropriate when two prior sequential results were both within the reference interval ([23](#_ENREF_23)) | 3,009 | 94,024 | 3.2 | BOYP |
| Sodium | Repeat was inappropriate when four or more sequential results were all within the reference interval ([23](#_ENREF_23)) | 3,214 | 100,453 | 3.2 | BOYP |
| Glucose | Repeat was inappropriate when four or more sequential results were all within the reference interval ([23](#_ENREF_23)) | 2,167 | 80,250 | 2.7 | BOYP |
| Sodium | Repeat was inappropriate when two prior sequential results were both within the reference interval ([23](#_ENREF_23)) | 2,712 | 100,453 | 2.7 | BOYP |
| Glucose | Repeat was inappropriate when two prior sequential results were both within the reference interval ([23](#_ENREF_23)) | 2,006 | 80,250 | 2.5 | BOYP |
| Total protein | Repeat was inappropriate when two prior sequential results were both within the reference interval ([23](#_ENREF_23)) | 483 | 37,132 | 1.3 | BOYP |
| Bilirubin | Repeat was inappropriate when four or more sequential results were all within the reference interval ([23](#_ENREF_23)) | 372 | 37,165 | 1.0 | BOYP |
| Alanine aminotransferase | Repeat was inappropriate when two prior sequential results were both within the reference interval ([23](#_ENREF_23)) | 305 | 38,090 | 0.8 | BOYP |
| Alkaline phosphatase | Repeat was inappropriate when two prior sequential results were both within the reference interval ([23](#_ENREF_23)) | 301 | 37,638 | 0.8 | BOYP |
| Alkaline phosphatase | Repeat was inappropriate when four or more sequential results were all within the reference interval ([23](#_ENREF_23)) | 226 | 37,638 | 0.6 | BOYP |
| Bilirubin | Repeat was inappropriate when two prior sequential results were both within the reference interval ([23](#_ENREF_23)) | 223 | 37,165 | 0.6 | BOYP |
| Gamma-glutamyltransferase | Repeat was inappropriate when two prior sequential results were both within the reference interval ([23](#_ENREF_23)) | 185 | 36,920 | 0.5 | BOYP |
| Total protein | Repeat was inappropriate when four or more sequential results were all within the reference interval ([23](#_ENREF_23)) | 186 | 37,132 | 0.5 | BOYP |
| Alanine aminotransferase | Repeat was inappropriate when four or more sequential results were all within the reference interval ([23](#_ENREF_23)) | 190 | 38,090 | 0.5 | BOYP |
| Aspartate aminotransferase | Repeat was inappropriate when two prior sequential results were both within the reference interval ([23](#_ENREF_23)) | 153 | 38,267 | 0.4 | BOYP |
| Gamma-glutamyltransferase | Repeat was inappropriate when four or more sequential results were all within the reference interval ([23](#_ENREF_23)) | 111 | 36,920 | 0.3 | BOYP |
| Aspartate aminotransferase | Repeat was inappropriate when four or more sequential results were all within the reference interval ([23](#_ENREF_23)) | 77 | 38,267 | 0.2 | BOYP |
| Albumin | Repeat was inappropriate when four or more sequential results were all within the reference interval ([23](#_ENREF_23)) | 44 | 44,212 | 0.1 | BOYP |
| Albumin | Repeat was inappropriate when two prior sequential results were both within the reference interval ([23](#_ENREF_23)) | 44 | 44,212 | 0.1 | BOYP |
| Complete blood count, prothrombin time, activated partial thromboplastin time, glucose, urea, creatinine, sodium, potassium, calcium, transaminases, gamma-glutamyltransferase, alkaline phosphatase, bilirubin, total protein, albumin, creatine kinase, lactate dehydrogonase, total cholesterol, LDL-cholesterol, HDL-cholesterol, triglycerides, uric acid, amylase and arterial blood gas | Inappropriate if test not relevant to symptoms/diagnosis, normal results not used to exclude diagnosis, repeated test not used for monitoring treatment, or test results made no difference to patient care ([24](#_ENREF_24)) | 11,048 | 24,428 | 45.2 | SOIP |
| Prothrombin time | Inappropriate if ordered to monitor patient receiving only low molecular weight or intravenous heparin ([6](#_ENREF_6)) | 117 | 232 | 50.4 | BONP |
| Partial thromboplastin time | Inappropriate if ordered to monitor patients receiving only low molecular weight heparin, only warfarin, or both ([6](#_ENREF_6)) | 115 | 232 | 49.6 | BONP |
| Venlafaxine | Inappropriate if repeated ≤2 days after change of dose (see Ref. Fig. 1 and Ref. 1) ([25](#_ENREF_25)) | 35 | 161 | 21.7 | BOIR |
| Venlafaxine | Inappropriate if repeated ≥6 days after change of dose (see Ref. Fig. 1 and Ref. 1) ([25](#_ENREF_25)) | 74 | 161 | 46.0 | BU |
| Mirtazapine, reboxetine, SSRIs, tricyclics | Inappropriate if repeated ≤4 days after change of dose (see Ref. Fig. 1 and Ref. 1) ([25](#_ENREF_25)) | 65 | 167 | 39.0 | BOIR |
| Mirtazapine, reboxetine, SSRIs, tricyclics | Inappropriate if repeated ≥8 days after change of dose (see Ref. Fig. 1 and Ref. 1) ([25](#_ENREF_25)) | 45 | 167 | 27.0 | BU |
| Prostate-specific antigen | Inappropriate if used to screen patients <40 years old or >75 years old for prostate cancer ([26](#_ENREF_26)) | 37,483 | 232,302 | 16.1 | BONP |
| Blood glucose | Inappropriate if both random and fasting glucose requested ([27](#_ENREF_27)) | 59 | 71,631 | 0.1 | BOYP |
| Complete blood count, electrolytes, urea, creatinine, prothrombin time, partial thromboplastin time, international normalized ratio, random blood glucose | In a pre-operative setting, inappropriate if not ordered in compliance with the pre-operative test guidelines of the National Institute for Health and Clinical Excellence (NICE) ([28](#_ENREF_28)) | 3 | 163 | 1.8 | BU |
| Complete blood count, electrolytes, urea, creatinine, prothrombin time, partial thromboplastin time, international normalized ratio, random blood glucose | In a pre-operative setting, inappropriate if ordered outside the pre-operative test guidelines of the National Institute for Health and Clinical Excellence (NICE) ([28](#_ENREF_28)) | 18 | 178 | 10.1 | BONT |
| *TPMT* genetic testing | Inappropriate if repeated ([5](#_ENREF_5)) | 253 | 7,710 | 3.3 | BOYP |
| *CYP2D6* genetic testing | Inappropriate if repeated ([5](#_ENREF_5)) | 4 | 433 | 0.9 | BOYP |
| *HFE* genetic testing | Inappropriate if repeated ([5](#_ENREF_5)) | 24 | 7,851 | 0.3 | BOYP |
| Amylase | In setting of acute pancreatitis, inappropriate if ordered in any circumstance other than on admission, following ERCP, or for recurrent symptoms ([29](#_ENREF_29)) | 162 | 227 | 71.4 | BONR |
| Alanine aminotransferase, alkaline phosphatase, total bilirubin, albumin | In setting of acute pancreatitis, inappropriate if ordered in any circumstance other than on admission, to formulate a modified Glasgow score, following ERCP, following surgery, or over 72 hours after a previous order (see Ref. Tables 2, 3) ([29](#_ENREF_29)) | 168 | 329 | 51.1 | BONR |
| Antineutrophil cytoplasmic antibody | Inappropriate unless for glomerulonephritis, pulmonary hemorrhage, cutaneous vasculitis, multiple lung nodules, chronic destructive disease of the airways, long-standing sinusitis or otitis, tracheal stenosis, peripheral neuropathy, or retro-orbital mass (see study’s ref. 1) ([30](#_ENREF_30)) | 376 | 1,127 | 33.4 | BOIR |
| Thyroid stimulating hormone, thyroxine, free thyroxine | In children with Down syndrome, inappropriate not to screen at 6 months, 12 months, and annually thereafter (see study Table 1 [Nebraska] and refs 8-9 from the American Association of Pediatrics) ([31](#_ENREF_31)) | 168 | 400 | 42.0 | BU |
| Thyroid stimulating hormone, thyroxine, free thyroxine | In children with Down syndrome, inappropriate not to screen at 6 months, 12 months, and annually thereafter (see study Table 1 [Oklahoma] and refs 8-9 from the American Association of Pediatrics) ([31](#_ENREF_31)) | 53 | 163 | 32.5 | BU |
| Erythrocyte sedimentation rate | In setting of suspected pulmonary embolism, inappropriate if ordered without evidence of previous abnormal values or worsening clinical indications ([32](#_ENREF_32)) | 53 | 126 | 42.1 | SOIP |
| Creatinine | In setting of suspected pulmonary embolism, inappropriate if ordered without evidence of previous abnormal values or worsening clinical indications ([32](#_ENREF_32)) | 127 | 379 | 33.5 | SOIP |
| Iron | In setting of suspected pulmonary embolism, inappropriate if ordered without evidence of previous abnormal values or worsening clinical indications ([32](#_ENREF_32)) | 22 | 77 | 28.6 | SOIP |
| Sodium | In setting of suspected pulmonary embolism, inappropriate if ordered without evidence of previous abnormal values or worsening clinical indications ([32](#_ENREF_32)) | 115 | 408 | 28.2 | SOIP |
| Blood urea nitrogen | In setting of suspected pulmonary embolism, inappropriate if ordered without evidence of previous abnormal values or worsening clinical indications ([32](#_ENREF_32)) | 28 | 103 | 27.2 | SOIP |
| Other hematology testing | In setting of suspected pulmonary embolism, inappropriate if ordered without evidence of previous abnormal values or worsening clinical indications ([32](#_ENREF_32)) | 115 | 493 | 23.3 | SOIP |
| White blood count, red blood count, coagulation studies | In setting of suspected pulmonary embolism, inappropriate if ordered without evidence of previous abnormal values or worsening clinical indications ([32](#_ENREF_32)) | 66 | 276 | 23.9 | SOIP |
| Potassium | In setting of suspected pulmonary embolism, inappropriate if ordered without evidence of previous abnormal values or worsening clinical indications ([32](#_ENREF_32)) | 96 | 414 | 23.2 | SOIP |
| Albumin | In setting of suspected pulmonary embolism, inappropriate if ordered without evidence of previous abnormal values or worsening clinical indications ([32](#_ENREF_32)) | 28 | 133 | 21.1 | SOIP |
| Glucose | In setting of suspected pulmonary embolism, inappropriate if ordered without evidence of previous abnormal values or worsening clinical indications ([32](#_ENREF_32)) | 62 | 307 | 20.2 | SOIP |
| Bilirubin | In setting of suspected pulmonary embolism, inappropriate if ordered without evidence of previous abnormal values or worsening clinical indications ([32](#_ENREF_32)) | 43 | 222 | 19.4 | SOIP |
| Alkaline phosphatase | In setting of suspected pulmonary embolism, inappropriate if ordered without evidence of previous abnormal values or worsening clinical indications ([32](#_ENREF_32)) | 37 | 194 | 19.1 | SOIP |
| Serum protein | In setting of suspected pulmonary embolism, inappropriate if ordered without evidence of previous abnormal values or worsening clinical indications ([32](#_ENREF_32)) | 25 | 134 | 18.7 | SOIP |
| Uric acid | In setting of suspected pulmonary embolism, inappropriate if ordered without evidence of previous abnormal values or worsening clinical indications ([32](#_ENREF_32)) | 36 | 198 | 18.2 | SOIP |
| Serum chloride | In setting of suspected pulmonary embolism, inappropriate if ordered without evidence of previous abnormal values or worsening clinical indications ([32](#_ENREF_32)) | 4 | 25 | 16.0 | SOIP |
| Gamma-glutamyltransferase | In setting of suspected pulmonary embolism, inappropriate if ordered without evidence of previous abnormal values or worsening clinical indications ([32](#_ENREF_32)) | 22 | 138 | 15.9 | SOIP |
| Aspartate aminotransferase | In setting of suspected pulmonary embolism, inappropriate if ordered without evidence of previous abnormal values or worsening clinical indications ([32](#_ENREF_32)) | 18 | 142 | 12.7 | SOIP |
| Calcium | In setting of suspected pulmonary embolism, inappropriate if ordered without evidence of previous abnormal values or worsening clinical indications ([32](#_ENREF_32)) | 17 | 139 | 12.2 | SOIP |
| Lactate dehydrogenase | In setting of suspected pulmonary embolism, inappropriate if ordered without evidence of previous abnormal values or worsening clinical indications ([32](#_ENREF_32)) | 15 | 133 | 11.3 | SOIP |
| Serum phosphate | In setting of suspected pulmonary embolism, inappropriate if ordered without evidence of previous abnormal values or worsening clinical indications ([32](#_ENREF_32)) | 6 | 68 | 8.8 | SOIP |
| Alanine aminotransferase | In setting of suspected pulmonary embolism, inappropriate if ordered without evidence of previous abnormal values or worsening clinical indications ([32](#_ENREF_32)) | 14 | 182 | 7.7 | SOIP |
| Ferritin | In setting of suspected pulmonary embolism, inappropriate if ordered without evidence of previous abnormal values or worsening clinical indications ([32](#_ENREF_32)) | 1 | 94 | 1.1 | SOIP |
| HIV Western blot | Inappropriate if repeat of a previous positive test ([33](#_ENREF_33)) | 19904 | 31504 | 63.2 | BONP |
| HIV, liver function tests, glucose, stool microscopy, blood smear, white blood count, urinalysis, pregnancy test, cerebrospinal fluid analysis, urine microscopy, syphilis testing (VDRL), ascites fluid analysis, pleural fluid analysis, lymph node biopsy/aspirate | Inappropriate if indicated based on specific national guidelines (see Ref. Table 1) but not ordered ([7](#_ENREF_7)) | 138 | 185 | 74.6 | SU |
| Hemoglobin | In Malawi, inappropriate if not ordered in setting of clinical pallor (see Ref. Table 1) ([7](#_ENREF_7)) | 10 | 43 | 23.3 | SU |
| Malaria smear | In Malawi, inappropriate if not ordered in setting of fever, rigors, chills in the absence of other obvious causes; in a <5 year-old, altered mental status, convulsions, extreme pallor and poor oral intake; or in adults, the above plus acute renal failure, acute respiratory distress syndrome, disseminated intravascular coagulopathy, jaundice, or hypoglycemia (see Ref. Table 1) ([7](#_ENREF_7)) | 43 | 117 | 36.8 | SU |
| Acid-fast bacteria sputum smear | Inappropriate if not performed in setting of chronic cough of more than three weeks’ duration (see Ref. Table 1) ([7](#_ENREF_7)) | 5 | 47 | 10.6 | SU |
| Blood transfusion/ crossmatch | Inappropriate if not ordered in case of severe anemia (Hb <4g/L), anemia (Hb <6 g/L) plus hemodynamic disturbance, acute hemorrhage plus shock, or intraoperatively when necessary ([7](#_ENREF_7)) | 6 | 19 | 31.6 | SU |
| Cerebrospinal fluid analysis, ascites analysis, pleural fluid analysis, lymph node biopsy | Cerebrospinal fluid analysis inappropriate absent suspected meningitis in the absence of clinically evident increased intracranial pressure; pleural fluid analysis inappropriate absent clinically significant pleural effusion; ascitic fluid analysis inappropriate absent clinically significant ascites; lymph node aspiration or biopsy inappropriate absent fluctuant/suppurative or solid lymphadenopathy (see Ref. Table 1) ([7](#_ENREF_7)) | 4 | 25 | 16.0 | SOIR |
| Hemoglobin | In Malawi, inappropriate absent clinical pallor (see Ref. Table 1) ([7](#_ENREF_7)) | 1 | 34 | 2.9 | SOIR |
| Urine microscopy, stool microscopy, urinalysis, white blood count with differential, VDRL, pregnancy test, liver function tests | Urine microscopy inappropriate absent suspicion of urinary tract infection or hematuria; stool microscopy inappropriate absent suspicion of diarrhea, anemia, or helminthic infection; urinalysis inappropriate absent urinary tract infection or generalized edema; white blood cell count with differential inappropriate absent fever; in setting of pregnancy, VDRL inappropriate as other than a screening test; pregnancy test inappropriate absent suspected pregnancy; liver function tests inappropriate absent suspected liver dysfunction (see Ref. Table 1) ([7](#_ENREF_7)) | 2 | 28 | 7.1 | SOIR |
| Malaria smear | In Malawi, inappropriate absent fever, rigors, or chills in the absence of other obvious causes; in a <5 year-old, altered mental status, convulsions, extreme pallor and poor oral intake; or in adults, the above plus acute renal failure, acute respiratory distress syndrome, disseminated intravascular coagulopathy, jaundice, or hypoglycaemia (see Ref. Table 1) ([7](#_ENREF_7)) | 5 | 79 | 6.3 | SOIR |
| Acid-fast bacteria sputum smear | Inappropriate absent chronic cough of more than three weeks’ duration (see Ref. Table 1) ([7](#_ENREF_7)) | 0 | 42 | 0.0 | SOIR |
| Amylase, lipase | Inappropriate absent suspicion of acute pancreatitis ([34](#_ENREF_34)) | 1,381 | 1,520 | 90.9 | SOYR |
| Alanine aminotransferase | Inappropriate if not measured prior to statin therapy initiation (see Ref. Table 1) ([35](#_ENREF_35)) | 2,577 | 5,717 | 44.1 | BU |
| Alanine aminotransferase | Inappropriate if not measured within 3-4 months of statin therapy initiation (see Ref. Table 1) ([35](#_ENREF_35)) | 3,098 | 3,637 | 85.2 | BU |
| HbSAg | In a pre-surgical screening setting, inappropriate if ordered ≤90 days after previous test ([36](#_ENREF_36)) | 538 | 3,480 | 15.5 | BOYP |
| D-dimer | In setting of suspected pulmonary embolism, inappropriate unless pre-test probability for pulmonary embolism is low (Well’s score ≤4); see Ref. Fig. 1 ([37](#_ENREF_37)) | 157 | 253 | 62.1 | BONR |
| All blood tests | Inappropriate if repeated within 12 hours of initial hospitalization but not clinically indicated ([38](#_ENREF_38)) | 6 | 268 | 2.2 | SOYP |
| Digoxin | Inappropriate unless in response to a change in toxicity-provoking physiologic parameters, such as decreased renal function; after addition or discontinuation of an interacting drug; to assess clinical response or adherence; or in presence of clinical signs of digoxin toxicity ([39](#_ENREF_39)) | 35 | 90 | 35.9 | SOIR |
| D-dimer | Inappropriate if D-dimer not performed before venous duplex scan in patient referred for imaging for suspected venous thromboembolism (VTE) but with low probability for same (no VTE risk factors); see Ref. Table 5 ([40](#_ENREF_40)) | 108 | 128 | 84.4 | BU |
| D-dimer | Inappropriate if D-dimer not performed before CT scan in patient referred for imaging for suspected venous thromboembolism (VTE) but with low probability for same (no VTE risk factors); see Ref. Table 5 ([40](#_ENREF_40)) | 24 | 64 | 37.5 | BU |
| D-dimer | Inappropriate if D-dimer not performed before V/Q scan in patient referred for imaging for suspected venous thromboembolism (VTE) but with low probability for same (no VTE risk factors); see Ref. Table 5 ([40](#_ENREF_40)) | 11 | 14 | 78.6 | BU |
| C-reactive protein | Inappropriate absent infection in patients with epistaxis ([41](#_ENREF_41)) | 25 | 28 | 89.3 | SOIR |
| Folate, ferritin, vitamin B12 | Inappropriate if repeat of a test ≤8 weeks earlier that was within the reference range and not borderline-low in patients ≥70 years old; see Ref. Fig. 1 ([42](#_ENREF_42)) | 157 | 2,598 | 6.0 | BOYP |
| Hemoglobin A1c | In setting of diabetes, inappropriate if repeated within 30 days of initial test ([43](#_ENREF_43)) | 11,003 | 130,538 | 8.4 | BOYP |
| Hemoglobin A1c | In setting of diabetes, inappropriate if repeated within 90 days of initial test ([43](#_ENREF_43)) | 40,162 | 130,538 | 30.8 | BOYP |
| Hemoglobin A1c | Inappropriate if not repeated within 365 days of initial test ([43](#_ENREF_43)) | 37,669 | 130,538 | 28.9 | BU |
| CD4 count | Inappropriate not to order in an HIV-positive patient if never before ordered, ordered only once ≥6 months ago, ordered only twice with ≥1<400 cells/µl ≥6 months ago, if previous result was <400 cells/µl ≥6 months ago, or no result in 12 months (see Ref. Table 1) ([44](#_ENREF_44)) | 253 | 436 | 58.0 | BU |
| CD4 count | Inappropriate not to order in an HIV-positive patient if never before ordered, ordered only once ≥6 months ago, ordered only twice with ≥1<400 cells/µl ≥6 months ago, if previous result was <400 cells/µl ≥6 months ago, or no result in 12 months (see Ref. Table 1) ([44](#_ENREF_44)) | 313 | 489 | 64.0 | BU |
| Lupus anticoagulant, anti-cardiolipin, anti-B2 glycoprotein antibodies | Inappropriate (“not justified”) unless for work-up of prolonged aPTT, thrombosis, or recurrent miscarriage for unknown reason or (“potentially justified”) in autoimmune disease ([45](#_ENREF_45)) | 96 | 520 | 18.5 | BONR |
| Protein C, Protein S, antithrombin | Inappropriate in setting of first venous thrombosis in patient >50 years old without family history; before diagnosis of venous thromboembolism was made; during acute thrombosis; during pregnancy or within 2 months of postpartum period; during warfarin therapy, heparin therapy; OCP use; specific other conditions (see Ref. Table 2) ([46](#_ENREF_46)) | 459 | 503 | 91.3 | BONT |

**Appendix Table 1. Study Measures of Inappropriate Testing, 1997-2012.** Measurements were extracted from studies as described in the main text. Note that in isolated cases, data were presented as per-patient percentages in the references, whereas here, as described in the main Methods section, they are presented as per test throughout. “Type” column is encoded as follows: B=objective, S=subjective; O=overutilization, U=underutilization; Y=repeat, N=non-repeat, I=both repeat and non-repeat; R=restrictive, P=permissive, T=both restrictive and permissive.

**Appendix Table 2. Overutilization and Underutilization over Time: Representative Analyses**

| **category** | **Analysis** | **bins** | **rates** | **direction** | **p-value** |
| --- | --- | --- | --- | --- | --- |
| overutilization | similar number of datapoints | 1997-2006 vs. 2007-2012 | 17.8 (12.2-23.4; n=66) vs. 24.3 (17.7-30.9); n=48 | increase | 0.139 |
|  | similar number of studies | 1997-2007 vs. 2008-2012 | 17.5 (12.0-23.0; n=68) vs. 25.0 (18.3-31.7; n=46) | increase | 0.089 |
|  | similar size bins | 1997-2004 vs. 2005-2012 | 39.1 (29.3-48.9; n=20) vs. 16.7 (12.3-21.1; n=94) | decrease | <0.001 |
|  | as above, sensitivity analysis (removing two biggest contributors to 2005-2012) | 1997-2004 vs. 2005-2012 | 39.3 (28.5-50.1; n=20) vs. 25.9 (19.4-32.4; n=53) | decrease | 0.039 |
| overutilization: initial | similar number of datapoints #1 | 1997-2005 vs. 2006-2012 | 38.5 (20.1-56.8; n=8) vs. 48.3 (31.9-64.8; n=10) | increase | 0.41 |
|  | similar number of datapoints #2 | 1997-2006 vs. 2007-2012 | 40.8 (24.2-57.4; n=10) vs. 47.9 (29.4-66.4; n=8) | increase | 0.55 |
| overutilization: repeat | similar number of datapoints #1 | 1997-2005 vs. 2006-2012 | 10.9 (4.2-17.5; n=17) vs. 5.8 (1.4-10.3; n=38) | decrease | 0.21 |
|  | similar number of datapoints #2 | 1997-2006 vs. 2007-2012 | 5.5 (1.5-9.4; n=45) vs. 16.0 (7.6-24.4; n=10) | increase | 0.027 |
| overutilization: objective | similar number of datapoints | 1997-2005 vs. 2006-2012 | 19.6 (11.2-28.2; n=25) vs. 14.3 (8.2-20.4; n=49) | decrease | 0.31 |
|  | as above, sensitivity analysis (removing single largest study) | 1997-2005 vs. 2006-2012 | 19.7 (10.5-28.9; n=25) vs. 30.6 (20.5-40.7; n=21) | increase | 0.116 |
| overutilization: subjective | no studies before 2002: no analysis |  |  |  |  |
| overutilization: restrictive | similar size bins #1 | 1997-2005 vs. 2006-2012 | 54.1 (37.7-70.5; n=12) vs. 36.8 (22.0-51.6; n=14) | decrease | 0.120 |
|  | similar size bins #2 | 1997-2006 vs. 2007-2012 | 50.6 (35.0-66.1; n=14) vs. 37.9 (21.4-54.2; n=12) | decrease | 0.26 |
| overutilization: permssive | similar size bins #1 | 1997-2005 vs. 2006-2012 | 10.2 (3.6-16.7; n=17) vs. 12.4 (9.0-15.8; n=65) | increase | 0.55 |
|  | similar size bins #2 | 1997-2006 vs. 2007-2012 | 7.8 (4.2-11.4; n=48) vs. 17.9 (13.5-22.3; n=34) | increase | 0.001 |
|  | as above, sensitivity analysis (removal of single study) | 1997-2006 vs. 2007-2012 | 15.7 (9.3-22.2; n=20) vs. 17.9 (13.5-22.3; n=34) | increase | 0.59 |
| underutilization | three bins | 2006-2008, 2009, 2011 | 24.7 (0-52.9; n=3) vs.  42.9 (26.4-59.4; n=9) vs. 58.0 (37.8-78.3; n=6) | increase | 0.151 |

**Supporting Information Table 2. Overutilization and Underutilization over Time: Representative Analyses.** Results of representative analyses, including sensitivity analyses, are summarized.

**S4.** References

1. van Walraven C, Naylor CD. Do we know what inappropriate laboratory utilization is?: A systematic review of laboratory clinical audits. JAMA 1998;280:550-8.

2. Begg CB, Mazumdar M. Operating characteristics of a rank correlation test for publication bias. Biometrics 1994;50:1088-101.

3. Isouard G. A quality management intervention to improve clinical laboratory use in acute myocardial infarction. Med J Aust 1999;170:11-4.

4. Bates DW, Boyle DL, Rittenberg E, Kuperman GJ, Ma'Luf N, Menkin V, et al. What proportion of common diagnostic tests appear redundant? Am J Med 1998;104:361-8.

5. Riegert-Johnson DL, Macaya D, Hefferon TW, Boardman LA. The incidence of duplicate genetic testing. Genet Med 2008;10:114-6.

6. Pilsczek FH, Rifkin WD, Walerstein S. Overuse of prothrombin and partial thromboplastin coagulation tests in medical inpatients. Heart Lung 2005;34:402-5.

7. Mepham SO, Squire SB, Chisuwo L, Kandulu J, Bates I. Utilisation of laboratory services by health workers in a district hospital in malawi. J Clin Pathol 2009;62:935-8.

8. Bates DW, Kuperman GJ, Rittenberg E, Teich JM, Fiskio J, Ma'luf N, et al. A randomized trial of a computer-based intervention to reduce utilization of redundant laboratory tests. Am J Med 1999;106:144-50.

9. Canas F, Tanasijevic MJ, Ma'luf N, Bates DW. Evaluating the appropriateness of digoxin level monitoring. Arch Intern Med 1999;159:363-8.

10. Rehmani R, Amanullah S. Analysis of blood tests in the emergency department of a tertiary care hospital. Postgrad Med J 1999;75:662-6.

11. Poteat HT, Chen P, Loughlin KR, Winkelman JW, Allada R, Ma'luf N, et al. Appropriateness of prostate-specific antigen testing. Am J Clin Pathol 2000;113:421-8.

12. Merlani P, Garnerin P, Diby M, Ferring M, Ricou B. Quality improvement report: Linking guideline to regular feedback to increase appropriate requests for clinical tests: Blood gas analysis in intensive care. BMJ 2001;323:620-4.

13. Mordasini MR, Krahenbuhl S, Schlienger RG. Appropriateness of digoxin level monitoring. Swiss Med Wkly 2002;132:506-12.

14. Huissoon AP, Carlton SA. Unnecessary repeat requesting of tests in a university teaching hospital immunology laboratory: An audit. J Clin Pathol 2002;55:78.

15. Affolter N, Krahenbuhl S, Schlienger RG. Appropriateness of serum level determinations of antiepileptic drugs. Swiss Med Wkly 2003;133:591-7.

16. Verstappen WH, van der Weijden T, Sijbrandij J, Smeele I, Hermsen J, Grimshaw J, Grol RP. Effect of a practice-based strategy on test ordering performance of primary care physicians: A randomized trial. JAMA 2003;289:2407-12.

17. Chen P, Tanasijevic MJ, Schoenenberger RA, Fiskio J, Kuperman GJ, Bates DW. A computer-based intervention for improving the appropriateness of antiepileptic drug level monitoring. Am J Clin Pathol 2003;119:432-8.

18. Loi S, Haydon AM, Shapiro J, Schwarz MA, Schneider HG. Towards evidence-based use of serum tumour marker requests: An audit of use in a tertiary hospital. Intern Med J 2004;34:545-50.

19. Ozbek OA, Oktem MA, Dogan G, Abacioglu YH. Application of hepatitis serology testing algorithms to assess inappropriate laboratory utilization. J Eval Clin Pract 2004;10:519-23.

20. Ramsey AH, Belongia EA, Chyou PH, Davis JP. Appropriateness of lyme disease serologic testing. Ann Fam Med 2004;2:341-4.

21. Walters RJ, Hutchings AD, Smith DF, Smith PE. Inappropriate requests for serum anti-epileptic drug levels in hospital practice. QJM 2004;97:337-41.

22. Kwok J, Jones B. Unnecessary repeat requesting of tests: An audit in a government hospital immunology laboratory. J Clin Pathol 2005;58:457-62.

23. Hawkins RC. Potentially inappropriate repeat laboratory testing in inpatients. Clin Chem 2006;52:784-5.

24. Miyakis S, Karamanof G, Liontos M, Mountokalakis TD. Factors contributing to inappropriate ordering of tests in an academic medical department and the effect of an educational feedback strategy. Postgrad Med J 2006;82:823-9.

25. Mann K, Hiemke C, Schmidt LG, Bates DW. Appropriateness of therapeutic drug monitoring for antidepressants in routine psychiatric inpatient care. Ther Drug Monit 2006;28:83-8.

26. Kerfoot BP, Holmberg EF, Lawler EV, Krupat E, Conlin PR. Practitioner-level determinants of inappropriate prostate-specific antigen screening. Arch Intern Med 2007;167:1367-72.

27. Wiwanitkit V, Lekngarm P. Requisition errors for blood glucose tests: A hospital-based study. Lab Medicine 2007;38:559-60.

28. Putnis S, Nanuck J, Heath D. An audit of preoperative blood tests. J Perioper Pract 2008;18:56-9.

29. Ward ST, Gilbert S, Mulchandani M, Garrett WV. Unnecessary investigations in patients with acute pancreatitis: Arterial blood sampling and serum biochemistry. Surgeon 2008;6:282-7.

30. Robinson PC, Steele RH. Appropriateness of antineutrophil cytoplasmic antibody testing in a tertiary hospital. J Clin Pathol 2009;62:743-5.

31. Fergeson MA, Mulvihill JJ, Schaefer GB, Dehaai KA, Piatt J, Combs K, et al. Low adherence to national guidelines for thyroid screening in down syndrome. Genet Med 2009;11:548-51.

32. Eva F, Rosanna Q, Laura C, Piero C, Silvio B. Appropriate laboratory utilization in diagnosing pulmonary embolism. Ann Clin Biochem 2009;46:18-23.

33. Hanna DB, Tsoi BW, Begier EM. Most positive hiv western blot tests do not diagnose new cases in new york city: Implications for hiv testing programs. J Acquir Immune Defic Syndr 2009;51:609-14.

34. Sutton PA, Humes DJ, Purcell G, Smith JK, Whiting F, Wright T, et al. The role of routine assays of serum amylase and lipase for the diagnosis of acute abdominal pain. Ann R Coll Surg Engl 2009;91:381-4.

35. Leaver H, Keng Lim T, Thomson P, Leaver J, Choy AM, Lang CC. Compliance to recommended liver function monitoring in patients on statin therapy. Cardiovasc Ther 2009;27:96-100.

36. Nies J, Colombet I, Zapletal E, Gillaizeau F, Chevalier P, Durieux P. Effects of automated alerts on unnecessarily repeated serology tests in a cardiovascular surgery department: A time series analysis. BMC Health Serv Res 2010;10:70.

37. Jones P, Elangbam B, Williams NR. Inappropriate use and interpretation of d-dimer testing in the emergency department: An unexpected adverse effect of meeting the "4-h target". Emerg Med J 2010;27:43-7.

38. Stewart BA, Fernandes S, Rodriguez-Huertas E, Landzberg M. A preliminary look at duplicate testing associated with lack of electronic health record interoperability for transferred patients. J Am Med Inform Assoc 2010;17:341-4.

39. Orrico KB, Wu M, Wilson AR. Assessment of the appropriateness of serum digoxin concentration measurement in a medical group setting. J Manag Care Pharm 2011;17:695-700.

40. Lee JA, Zierler BK. The current state of practice in the diagnosis of venous thromboembolism at an academic medical center. Vasc Endovascular Surg 2011;45:22-7.

41. Biggs TC, Nightingale K. Inappropriate c-reactive protein testing in epistaxis patients. Clin Otolaryngol 2011;36:405-6.

42. Ganiyu-Dada Z, Bowcock S. Repeat haematinic requests in patients with previous normal results: The scale of the problem in elderly patients at a district general hospital. Int J Lab Hematol 2011;33:610-3.

43. Laxmisan A, Vaughan-Sarrazin M, Cram P. Repeated hemoglobin a1c ordering in the va health system. Am J Med 2011;124:342-9.

44. Were MC, Shen C, Tierney WM, Mamlin JJ, Biondich PG, Li X, et al. Evaluation of computer-generated reminders to improve cd4 laboratory monitoring in sub-saharan africa: A prospective comparative study. J Am Med Inform Assoc 2011;18:150-5.

45. Mameli A, Barcellona D, Vannini ML, Marongiu F. High frequency of inadequate test requests for antiphospholipid antibodies in daily clinical practice. Clin Chem Lab Med 2011;49:695-8.

46. Tientadakul P, Chinthammitr Y, Sanpakit K, Wongwanit C, Nilanont Y. Inappropriate use of protein c, protein s, and antithrombin testing for hereditary thrombophilia screening: An experience from a large university hospital. Int J Lab Hematol 2011;33:593-600.

1. The difference between this number and the 25,489 referenced in Fig. 1 are 762 citations for which a Medline update was in progress when this analysis was performed. [↑](#footnote-ref-1)
2. Excluding from the 493 studies included for in-depth review the four studies that could not be found. [↑](#footnote-ref-2)
